# Systematic analysis reveals a functional role for STAMBPL1 in the epithelial–mesenchymal transition process across multiple carcinomas

**DOI:** 10.1038/s41416-020-0972-x

**Published:** 2020-07-08

**Authors:** Gorbatchev Ambroise, Ting-ting Yu, Boxi Zhang, Merve Kacal, Yuqing Hao, Andre L. Queiroz, Amanda T. Ouchida, Cecilia Lindskog, Erik Norberg, Helin Vakifahmetoglu-Norberg

**Affiliations:** 1 Department of Physiology and Pharmacology, Karolinska Institutet, Solnavägen 9, Biomedicum, 17165 Stockholm, Sweden; 2 Department of Medical Genetics, School of Basic Medical Sciences, Nanjing Medical University, Nanjing, Jiangsu 211166 PR China; 3 Department of Immunology, Genetics and Pathology, Rudbeck Laboratory, Uppsala University, 75185 Uppsala, Sweden

**Keywords:** Cancer, Cell biology

## Abstract

**Background:**

Deubiquitinating enzymes (DUBs) are linked to cancer progression and dissemination, yet less is known about their regulation and impact on epithelial–mesenchymal transition (EMT).

**Methods:**

An integrative translational approach combining systematic computational analyses of The Cancer Genome Atlas cancer cohorts with CRISPR genetics, biochemistry and immunohistochemistry methodologies to identify and assess the role of human DUBs in EMT.

**Results:**

We identify a previously undiscovered biological function of STAM-binding protein like 1 (STAMBPL1) deubiquitinase in the EMT process in lung and breast carcinomas. We show that STAMBPL1 expression can be regulated by mutant p53 and that its catalytic activity is required to affect the transcription factor SNAI1. Accordingly, genetic depletion and CRISPR-mediated gene knockout of STAMBPL1 leads to marked recovery of epithelial markers, SNAI1 destabilisation and impaired migratory capacity of cancer cells. Reversely, STAMBPL1 expression reprogrammes cells towards a mesenchymal phenotype. A significant STAMBPL1-SNAI1 co-signature was observed across multiple tumour types. Importantly, STAMBPL1 is highly expressed in metastatic tissues compared to matched primary tumour of the same lung cancer patient and its expression predicts poor prognosis.

**Conclusions:**

Our study provides a novel concept of oncogenic regulation of a DUB and presents a new role and predictive value of STAMBPL1 in the EMT process across multiple carcinomas.

## Background

The ubiquitin-proteasome system (UPS) involves the recognition and targeting of proteins to the proteasome for their substrate-specific protein degradation.^1,2^ While ubiquitination conjugation processes covalently attaches ubiquitin, deubiquitinating enzymes (DUBs) remove ubiquitin moieties from target proteins.^3–6^ Based on sequence similarity and catalytic domains, human DUBs are divided into subclasses, and to maintain a high level of specificity, DUBs deal with ubiquitin chains of distinct topology, length and linkage type.^7,8^ Accordingly, deubiquitination of different chain linkages affects substrates in distinct cellular functions and multiple biological processes. Moreover, by controlling the stability and abundance of pathological proteins, DUBs have been predicted to be linked to human diseases, including progressive carcinogenesis.^5,9^ Correspondingly, growing evidence indicates DUBs to be mutated or altered in expression in various cancer cells and tumours, suggesting their roles as oncogenes and tumour suppressors.^9–11^ Nevertheless, many human DUBs are still poorly understood when it comes to their cellular function, targets and regulation.

Cancer cells elevate the level of their malignancy by undergoing a fundamental process called epithelial–mesenchymal transition (EMT),^12^ which allows epithelial cells to undergo multiple reversible biochemical changes that enable them to adopt a mesenchymal phenotype by altering the expression of proteins in cell polarity, cell–cell contact and extracellular matrix. Consequently, cells undergoing EMT become motile and invasive by acquiring fibroblast-like properties beyond displaying key mesenchymal hallmarks characterised by the loss of epithelial integral membrane proteins, such as E-cadherin (*CDH1*) and Claudin-1, while concomitantly upregulating intermediate filament and adhesion proteins, including vimentin (*VIM*) and N-cadherin.^10,13–15^ However, EMT can also give rise to a variety of intermediate cell states, which may sustain signals and expression of markers of both epithelial and mesenchymal state.^16^

The transdifferentiation to a mesenchymal-like state is driven by a network of key EMT-inducing transcription factors (TFs), including SNAI1 (Snail), SNAI2 (Slug), ZEB1 and TWIST,^12,17^ that are tightly regulated by ubiquitin modifications. The aberrant expression of these TFs has been frequently observed to correlate with poor prognosis in many types of cancer.^18^ Given that EMT-TFs are subjected to degradation by the UPS, DUBs are emerging as potential new regulators of the EMT process.^10,12^ Additionally, as increasing data propose DUBs to be commonly altered in human cancers,^5,9,12,19,20^ they represent attractive targets for drug discovery for cancer therapeutics. However, the regulation of DUBs under carcinogenic conditions is largely unexplored. While DUB regulatory mechanisms include cellular localisation and catalytic activity, their abundances can be regulated in specific, context-dependent manner to ensure channelling of the appropriate cellular responses.^12,21^ In fact, the role and expression profile of DUBs can differ in different tumour types,^10^ which may depend on the genetic background of the tumour. Yet, whether oncogenes promoting cell migration, invasion and EMT might regulate the DUB abundance and if such interaction would be relevant for the EMT process remain elusive.

Here, we undertook an integrative approach to uncover DUBs involved in the EMT process by a systematic correlation screening of all human DUBs in lung and breast tumour biopsies, representing the two most common cancer diseases worldwide. Beyond providing previously unknown new function of STAM-binding protein like 1 (STAMBPL1) in the EMT program, our work presents a new concept of oncogenic regulation of STAMBPL1 by mutant p53. Based on our combinatorial study comprising functional cancer genomics, human cancer specimens and biochemical approaches, we propose STAMBPL1 as a potential new predictive signature of EMT, and present a therapeutic opportunity of targeting STAMBPL1 to exhaust the EMT potential of cancer cells.

## Methods

### Analysis of TCGA LUAD and BRCA cancer cohorts

Illumina HiSeqV2, RSEM (RNA-sequencing by expectation-maximisation) normalised RNA-seq gene expression data of 517 lung adenocarcinoma (LUAD) and 1100 breast invasive carcinoma (BRCA) tumour samples were retrieved from the Broad Firebrowse The Cancer Genome Atlas (TCGA) (data version 2016_01_28; http://firebrowse.org/). The Pearson correlation between DUB genes and vimentin (*VIM*) were computed using R package Hmisc (https://CRAN.R-project.org/package=Hmisc). DUB genes that positively correlated to *VIM* with correlation coefficient >0.2 were further analysed by the same package to compute the Pearson correlation matrix with both *VIM* and E-cadherin (*CDH1*). The correlation matrix was then visualised via the corrplot package (https://github.com/taiyun/corrplot).

### Analysis of 37 TCGA tumour types

The Illumina HiSeqV2, RSEM normalised RNA-seq gene expression data of *STAMBPL1* and *SNAI1* in 37 different cancer cohorts were retrieved from TCGA Research Network: http://cancergenome.nih.gov/ using the TCGA2STAT package. The data were log 2 transformed and based on the TCGA barcode, only the data from tumour samples were kept for further analysis. Pearson correlation analysis was performed to assess the co-expression between the gene expression of *STAMBPL1* and *SNAI1* across all 37 cancer types. A correlation was considered as significant in a cancer if the Benjamini–Hochberg adjusted *P* < 0.01.

### EMT metric

The EMT scores were calculated using an EMT prediction model,^22^ based on a 76-gene expression signature.^23^ For each LUAD and BRCA patient sample, the EMT score was calculated as a weight sum of 76 gene expression levels using the equation $\mathop {\sum}\nolimits_{i = 1}^{76} {w_iG_{ij}}$, where *w*_*i*_ is the correlation coefficient between the *i*th gene expression from the 76 genes and that of E-cadherin (*CDH1*), and *G*_*ij*_ is the *i*th gene’s normalised log 2 expression level in the *j*th sample. The scores were then centred by subtracting the mean across all tumour samples resulting in the grand mean score to be zero. Based on this model, positive value scores (>0) correspond to the epithelial phenotype, whereas negative value scores (<0) reflect mesenchymal phenotype, as an EMT scoring metric.

### Analysis of CCLE cell lines

The RNA-seq data of *STAMBPL1*, *SNAI1*, *VIM*, and *CDH1* for different LUAD (*n* = 50) and breast carcinoma cell lines (n = 57) were downloaded from Broad Institute Cancer Cell Line Encyclopaedia (CCLE) (https://portals.broadinstitute.org/ccle). The Pearson correlation analysis was then performed between the gene expression data of STAMBPL1 and the genes of interest, respectively. The correlation coefficient was calculated and the significance was set as *p* value < 0.05.

### Kaplan–Meier analysis

According to the TCGA clinical data for LUAD that was retrieved from Broad Firebrowse, a total of 492 patients who express STAMBPL1 were included in the Kaplan–Meier analyses. While the performance of all possible cut-off values between the upper and lower quantiles were calculated based on log-rank test using the survival package, the best-performing threshold was used as a cut off to determine the survival in the low- and high-risk groups.

### Immunohistochemical analysis of primary and metastatic lung cancer tissues

Immunohistochemistry and slide scanning were performed as previously described.^24,25^ Consecutive microarray sections of primary and metastatic lung cancer tissues were purchased from Biocat. The sections were deparaffinised in xylene, hydrated in graded alcohols and blocked for endogenous peroxidase in 0.3% hydrogen peroxide diluted in 95% ethanol. For antigen retrieval, a Decloaking chamber (Biocare Medical) was used. Immunohistochemical staining of vimentin, E-cadherin and STAMBPL1 was performed using an Autostainer 480 instrument (Thermo Fisher Scientific), incubating the slides with anti-vimentin (HPA001762, Atlas Antibodies Ab, diluted 1:1000), anti-E-cadherin (HPA004812, Atlas Antibodies Ab, diluted 1:300), anti-p53 (CAB002973, diluted 1:1000) and anti-STAMBPL1 (HPA040202, Atlas Antibodies AB, diluted 1:600) for 30 min. The slides were incubated with secondary reagent anti-rabbit/mouse horseradish peroxidase-conjugated UltraVision (Thermo Fisher Scientific) for 30 min, and developed for 10 min using diaminobenzidine Quanto (Thermo Fisher Scientific) as chromogen. All incubations were followed by rinsing in wash buffer (Thermo Fisher Scientific) 2 × 5 min. Slides were counterstained in Mayers haematoxylin (Histolab) and cover slipped using Pertex (Histolab) as mounting medium. The stained slides were digitalised using the automated scanning system Aperio AT2 (Aperio Technologies), using a ×20 objective.

### CRISPR/Cas9-mediated genome editing

Single guide RNAs (sgRNAs) for gene-specific targeting were designed using the online tool http://crispr.mit.edu website. The targeting RNP complex, composed of synthetic Alt-RCRISPR-Cas9 sgRNA (IDT) and Alt-R S.p. HiFi Cas9 Nuclease V3 (IDT), was delivered into NCI-H838 cells by Lipofectamine RNAiMAX (Invitrogen). After transfection, single-cell clones were collected and cultured for genotyping by PCR to confirm the positive clone with edited STAMBPL1. Sequences are stated in the Supplementary information.

### siRNAs and plasmids

Forty nanomolar small interfering RNA (siRNA) targeting *STAMBPL1*, *UbB* and *UbC* and non-targeting (N.T.) siRNA was used, and the transfection was performed using Lipofectamine 2000 Reagent (Invitrogen) according to the manufacturer’s instructions. The knockdown efficiency was assessed by Western blotting 48–72 h post transfection. siRNA sequences are stated in the Supplementary information. Transient expression transfections were performed with Lipofectamine 2000 Reagent (Invitrogen) or ViaFect Reagent (Promega), as recommended by the manufacturer, and protein expression for 24–48 h was allowed for before experimental procedures. Cells were transfected with 1–2 µg plasmid for the expression of green fluorescence protein as a control, pCMV6- SNAIL (SNAI1) STAMBPL1 (STAMBPL1-Myc-DDK, RC201884) from OriGene or STAMBPL1 D360A mutant in the active site. MCF-10A cells stably expressing an empty vector (PCB6+) or constructs expressing p53175H (in PCB6+; 72R polymorphism) with effectene reagent (Qiagen) were created as previously described.^26^

#### For site-directed mutagenesis

D360A mutation in STAMBPL1-Myc-DDK construct was generated by site-directed mutagenesis using QuikChange II XL Site-Directed Kit (Agilent Technologies). Oligo sequences are stated in the Supplementary information. Mutagenesis was verified using sequencing.

### Chromatin immunoprecipitation coupled with quantitative PCR (ChIP-qPCR)

ChIP was performed using the EpiQuik ChIP Kit (Epigentek, P-2002-2). MCF-10A cells (untreated or MG132 (2 µM)-treated p53^WT^ MCF-10A cells and mutant p53^R175H^-stable MCF-10A cells) were fixed with 1% formaldehyde for 10 min. Crosslinking was stopped by adding 125 mM glycine. Then, formaldehyde-crosslinked chromatin was sheared with Bioruptor® Sonicator into short fragments between 200 and 1000 bp in size. p53-bound chromatin segments were immunoprecipitated with 3 μl of anti-p53 monoclonal (DO-1) antibody (Santa Cruz, sc-126). ChIP samples were analysed by using SYBR Green qPCR Master Mix.

### Ubiquitination and in vitro DUB assay

The 293T cells were transfected with 1 μg of Snail, HA-ubiquitin and WT STAMBPL1 or D360A STAMBPL1 plasmids. Lysates were subjected to immunoprecipitation (IP) with anti-Snail and immunoblotting with anti-ubiquitin to examine ubiquitination level of Snail. For in vitro DUB assay: Deubiquitination cleavage assays were performed using recombinant GST-tagged STAMBPL1 (Ubiquigent) and polyubiquitin chains (BostonBiochem). STAMBPL1 were freshly prepared in assay buffer (40 mM Tris-Hcl, pH 7.5, 5 mM dithiothreitol (DTT), 0.005% bovine serum albumin) and activated for 10 min at 30 °C. Subsequently, 1 μg of STAMBPL1 were incubated with 200 ng of either Lys^63^ or Lys^48^ polyubiquitin (Ubi_1_–Ubi_7_) chains at 30 °C for the indicated time points. The reactions were stopped by the addition of SDS sample buffer (Bio-Rad) supplemented with 100 mM freshly prepared DTT). Ubiquitin cleavage was detected using immunoblotting and anti-ubiquitin antibodies (Enzo, BML-PW8810).

### Statistics

Statistical analysis was performed using R (version 3.6.1) and GraphPad Prism (version 8.2.1). For all experiments with error bars, standard deviation (SD) was calculated and *p* values represent mean ± SD. Presented data are an average of at least three independent experiments (*n* ≥ 3) or representative of independent experiments.

## Results

### Identification of deubiquitinases associated with mesenchymal markers in human lung and breast cancer

**Fig. 1:**
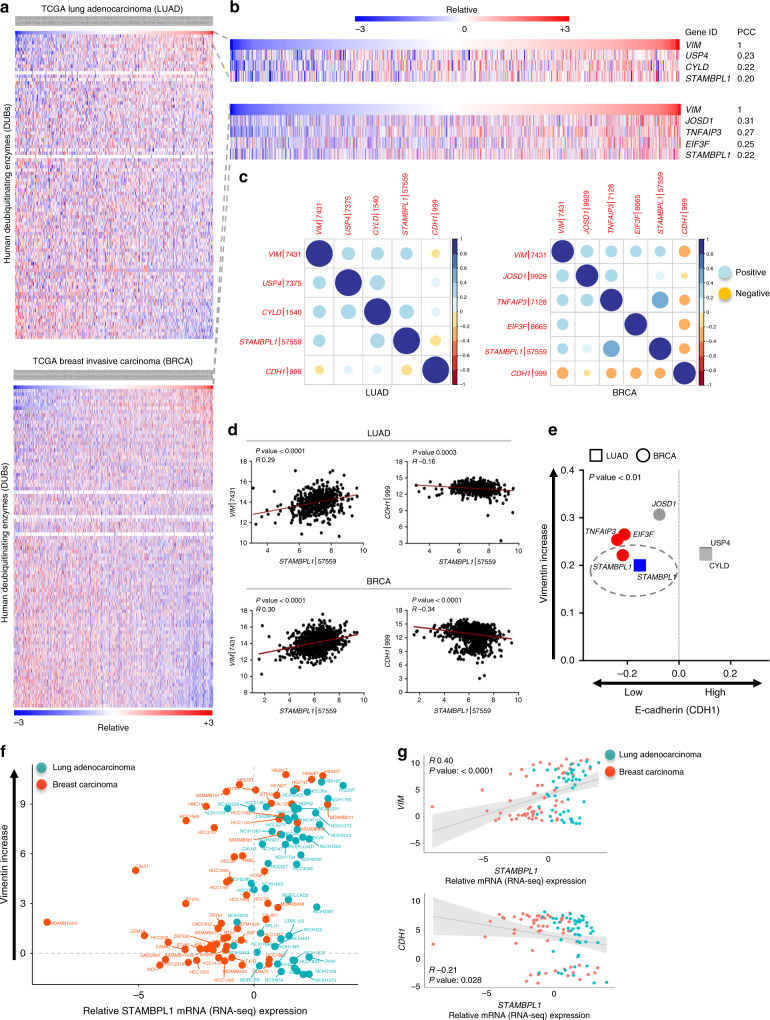
Identification of human DUBs correlated with mesenchymal phenotype in tumour biopsies. **a** Heat map of hierarchical clustering of all human deubiquitinases (DUBs) with *VIM* expression in normalised RNA-seq data across LUAD and BRCA tumour biopsies. Each row represents a DUB gene and each column represents a tumour sample. **b** Top correlated DUB genes clustering with *VIM* inset with indicated Pearson correlation coefficient (PCC ≥ 0.2) highlighted in a zoom-up view. **c** Pearson correlation matrix (*p* < 0.05) of the EMT markers *CDH1* and *VIM*, and the top correlated DUBs highlighted for both LUAD (left panel) and BRCA (right panel). Positive correlations are displayed in blue and negative correlations in yellow colour. Intensity and the size of the circle are proportional to the correlation coefficients. **d** Linear regression analysis between the gene expression level of *STAMBPL1* and *VIM* or *CDH1* for LUAD (top two panels) and BRCA (bottom two panels). **e** Scatter plot showing the *CDH1* and *VIM* expression levels in tumour biopsies exhibiting high expression of the indicated DUBs. Significantly correlated genes (*p* < 0.01) are depicted in colour (blue for LUAD and red for BRCA). Not significant was indicated in grey. **f**, **g** Tumour genomic RNA-seq expression data from 107 human lung and breast cancer cell lines analysed for expression correlation of *STAMBPL1* with *VIM* or *CDH1* using the CCLE dataset.

To systematically identify the potential DUB contribution to the EMT process, we reasoned that correlative DUBs should be co-elevated with high expression of classical mesenchymal markers. To this end, the expression of the intermediate filament protein, vimentin (*VIM*), characteristically upregulated in cells during EMT,^15^ was correlated with the expression of all defined human DUBs,^27^ in normalised RNA-seq data of 517 lung adenocarcinoma (LUAD) and 1100 Breast invasive carcinoma (BRCA) tumour biopsies retrieved from The Cancer Genome Atlas (TCGA) Research Network: http://cancergenome.nih.gov/ (Fig. 1a). Pearson correlation coefficient (PCC) of the top significant hits (PCC ≥ 0.2) revealed a small number of cancer-type-specific DUBs, including USP4, previously shown to play a critical role in regulating EMT.^10^ One DUB, coincided in both LUAD and BRCA cohorts, displaying a significant positive correlation (PCC ≥ 0.2 and *p* < 0.05) with high *VIM* levels (Fig. 1b). Further the expression of the top significant DUBs in both cancer types was simultaneously inversely correlated with the expression of an additional molecular marker, E-cadherin (*CDH1*), whose loss is considered to be fundamental for EMT. This combined criterion allowed a more stringent approach and increased the probability to identify the DUBs that are clinically relevant across multiple tumour types (Fig. 1c). These systematic co-expression correlation analyses revealed STAMBPL1/AMSH-LP, a member of the JAMM metalloprotease family, among the top candidate DUBs with a significant (*p* < 0.01) positive correlation with *VIM*, while a concurrent significant (*p* < 0.01) negative correlation with *CDH1* in both cancer types (Fig. 1d, e).

Additionally, a quantification method to investigate the correlation of STAMBPL1 expression with an EMT metric using a prediction model was applied to the LUAD and BRCA cohorts. For each patient sample (>1600), we calculated the EMT score that predicts either a positive value (>0) corresponding to the epithelial phenotype or negative value scores (<0) reflecting mesenchymal phenotype, as an EMT scoring metric. This algorithm found that *STAMBPL1* expression level was negatively correlated in LUAD (*r* −0.22, *p* < 0.0001) and in BRCA (*r* −0.37, *p* < 0.0001) to the EMT scores (Supplementary Fig. S1a), which further confirm STAMBPL1 to be significantly associated with mesenchymal traits in tumours biopsies, thus focused on in this study.

Carcinomas often display heterogeneity in epithelial and mesenchymal markers in various combinations.^16^ This necessitated us to interrogate whether the clinical patient data on STAMBPL1 translate into the genomic features of carcinoma cell line models. Therefore, we retrieved data on detailed integrative genomics of a large panel cell lines from the CCLE.^28,29^ Tumour genomic RNA-seq expression data from 107 human lung adenocarcinoma and breast ductal carcinoma cell lines were analysed for expression correlation of *STAMBPL1* with *VIM* (Fig. 1f). Although carcinomas rarely execute a complete EMT program, which hinders them to fully display a mesenchymal state, the cell line analyses uncovered a significant positive co-expression profile of *STAMBPL1* with *VIM* with a simultaneous significant (*p* < 0.05) negative correlation with *CDH1* in cells originating from both tissue types (Fig. 1g). This observation confirms that the identified STAMBPL1 signature in primary tumour biopsies is reflected in cancer cell lines, and signify that discoveries on molecular mechanism involving STAMBPL1 in cancer cells, can serve as a basis for the biological interpretation of EMT program in human cancer.

### STAMBPL1 depletion affects the mesenchymal phenotype in lung and breast cancer cells

**Fig. 2:**
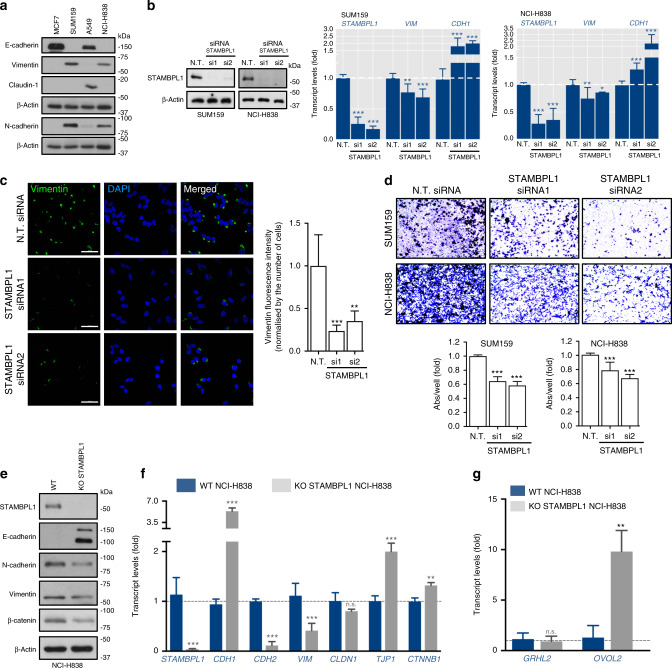
STAMBPL1 depletion affects EMT and cell migration in lung and breast cancer cells. **a** Western blot of indicated epithelial and mesenchymal marker proteins in breast (SUM159 and MCF7) and lung (NCI-H838 and A549) cancer cells. β-Actin is shown twice due to that E-cadherin and N-cadherin were stained separately on the same gel. **b** Relative mRNA levels of *VIM* and *CDH1* in SUM159 and NCI-H838 cells transfected for 48 h with an N.T. or siRNA targeting STAMBPL1. The siRNA knockdown efficiency is shown both at mRNA (qPCR) and protein levels (Western blot) in SUM159 and NCI-H838 cells. **c** Immunofluorescence staining of vimentin and DAPI in SUM159 cells, transfected for 48 h with an N.T. or STAMBPL1 siRNAs. Scale bar, 50 μm. Quantification of vimentin signal/nuclei (>50 cells per condition per replicate) (*n* = 6). **d** Transwell migration assay in cells transfected for 48 h with N.T. or siRNA targeting STAMBPL1. Quantification is presented as fold over control bar graphs (*n* = 3). **e**, **f** Protein (Western blot) and relative mRNA (qPCR) levels of indicated epithelial and mesenchymal marker genes in control WT or STAMBPL1 KO NCI-H838 cells. **g** Relative mRNA levels of *GRHL2* and *OVOL2* in STAMBPL1 WT and KO NCI-H838 cells. Statistical significance is shown over each control. *p < 0.05, **p < 0.001 and ***p < 0.0001 (Student’s *t* test). Data are presented as mean ± SD (*n* ≥ 3). β-Actin was shown as equal loading for all immunoblots. n.s. not significant.

To experimentally investigate the potential role of STAMBPL1 in the EMT process, we used the lung adenocarcinoma (A549, NCI-H838) and breast carcinoma (MCF7, SUM159) cell lines, displaying either epithelial or mesenchymal traits, respectively. Compared to the NCI-H838 cells that are derived from lymph node metastasis, A549 are epithelial cells isolated from adenocarcinoma human alveolar that have not spread to the nearby lymph node. Accordingly, NCI-H838 cells displayed higher levels of vimentin and N-cadherin expression concomitant with significant low E-cadherin and claudin-1 levels (Fig. 2a). Consistently, SUM159, which is a claudin-low triple-negative breast cancer cell line, displayed high levels of vimentin and N-cadherin, along with a loss of E-cadherin expression, compared to MCF7, a widely studied epithelial cell line derived from breast adenocarcinoma (Fig. 2a). Thus, in both NCI-H838 and SUM159 cells, concomitant changes in expression of EMT marker proteins were detected to coincide with the mesenchymal status of these cancer cells. Next, we analysed the effect of genetic depletion of STAMBPL1 by two independent siRNAs in these cell lines (Fig. 2b). qPCR results revealed, beyond the knockdown efficiency, that depletion of STAMBPL1 causes an elevation of *CDH1* and a reduction of *VIM* messenger RNA (mRNA) levels (Fig. 2b). In line with this, the suppressive effect of STAMBPL1 knockdown on vimentin was confirmed by immunofluorescence staining of NCI-H838 cells (Fig. 2c). Further, as EMT is strongly implicated in greater invasive abilities of tumour cells, it predicts that silencing of STAMBPL1 should cause suppressive effects on cell migration. Consistently, SUM159 and NCI-H838 cancer cells became less migratory upon STAMBPL1 depletion as observed by transwell migration and wound scratch assays (Fig. 2d and Supplementary Fig. S1b). Since cells’ migratory capacity may reflect cell growth, we performed cell proliferation assays and found that the proliferative ability of the cells was unaffected upon STAMBPL1 depletion (Supplementary Fig. S1c). These results suggest that STAMBPL1 depletion leads to significant molecular changes of EMT markers as well as the phenotypic readouts.

To further validate the molecular effects of STAMBPL1 on EMT, we used CRISPR/Cas9 genome editing approach to generate a STAMBPL1 gene knockout (KO) in NCI-H838 cancer cells (Supplementary Fig. S2a), in which we assessed both the mRNA and protein level of EMT markers, including N-cadherin (*CDH2*), Claudin-1 (*CLDN1*), ZO-1 (*TJP1*), *β*-catenin (*CTNNB1*) beyond E-cadherin (*CDH1*) and vimentin (*VIM*) (Fig. 2e, f). These analyses showed that the KO of STAMBPL1 caused a significant restoration in the expression of E-cadherin, both transcriptionally and at protein levels, and a significant vimentin reduction. The transcriptional upregulation of the epithelial marker ZO-1 was also significant in the KO cells with a concomitant marked decrease in N-cadherin levels, which was correspondingly mirrored by the detected reduction of its protein (Fig. 2e, f). Contrary to these EMT markers, no major changes were seen on the Claudin-1 mRNA levels, whereas *β*-catenin showed opposing expression alterations. While a decrease on its protein expression was noticed, the *CTNNB1* transcription expression displayed a slight increase (Fig. 2e, f), which may be able to compensate the decrease in protein load.

Due to its considerable effect on E-cadherin (*CDH1*) re-expression, we analysed if STAMBPL1 KO correspondingly could trigger the activation of EMT inhibitors, including *GRHL2* and *OVOL2*, both of which are among the transcriptional regulators of *CDH1*. While no changes were observed in *GRHL2*, the KO of STAMBPL1 lead to a substantial increase in the transcript levels of *OVOL2* (Fig. 2g). Collectively, these data support, beyond a significant correlation between mesenchymal phenotypes with STAMBPL1, that STAMBPL1 depletion cause significant molecular changes of EMT.

Furthermore, consistent with the knockdown studies (Supplementary Fig. S1c), KO of STAMBPL1 showed no effect on NCI-H838 cell proliferation compared to the WT cells (Supplementary Fig. S1d).

### STAMBPL1 expression contributes to EMT

**Fig. 3:**
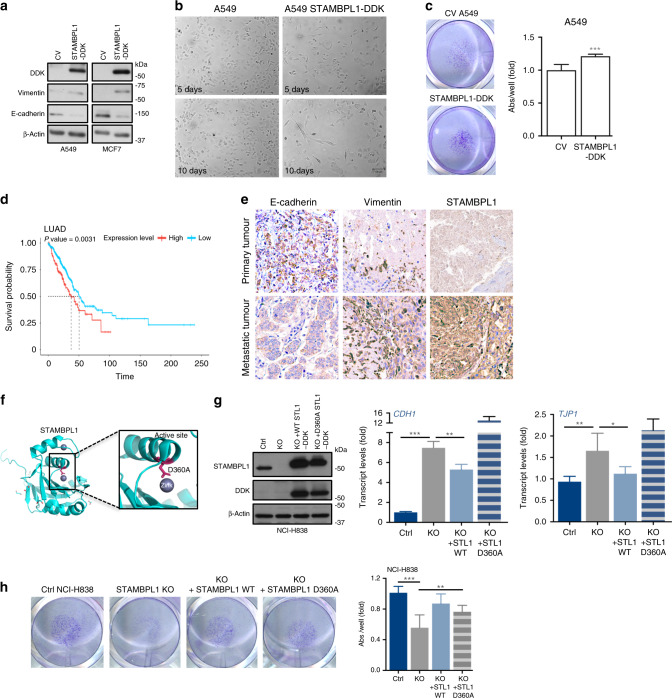
STAMBPL1 expression promotes EMT and cell migration. **a** Western blots of DDK, E-cadherin and vimentin in A549 and MCF7 cells transfected for 48 h with a control vector (CV) or WT STAMBPL1-DDK plasmid. **b** Morphology of control and A549 cells expressing WT STAMBPL1-DDK up to 10 days (normal light, magnification ×20, scale bar 100 μm). **c** Transwell migration assay of A549 cells expressing a control or WT STAMBPL1-DDK plasmids. Quantification is presented as fold over control (*n* = 3). **d** Kaplan–Meier analysis and overall survival of high or low STAMBPL1 expressing 492 lung adenocarcinoma patients. **e** Tissue from primary and metastatic lung tumours originating from the same patient were immunostained for STAMBPL1, vimentin or E-cadherin expression. **f** Model structure of STAMBPL1 protein (PDB: 2ZNR^34^). The STAMBPL1-Jab1/MPN domain structure is shown with the zinc (in purple) coordinating residue mutated to generate a catalytic inactive STAMBPL1 (Asp360: pink, D360A). A zoom-up view of the interaction is shown in the inset. **g** (left panel) Western blots showing the expression of DDK-tagged WT or mutant D360A STAMBPL1. (Right panels) Relative mRNA (qPCR) levels of *CDH1* and *TJP1* in control, STAMBPL1 KO NCI-H838 and in KO cells subsequent to exogenous expression of the WT or D360A-STAMBPL1. **h** Transwell migration assay of cells NCI-H838 cells as in **g**. Quantification is presented as fold over control (*n* = 3). β-Actin was shown as equal loading. Data are presented as mean ± SD (*n* = 3). Statistical significance is shown over the control, or as shown otherwise. *p < 0.05, **p < 0.001 and ***p < 0.0001 (Student’s *t* test).

We then probed the ability of STAMBPL1 to reprogramme and induce mesenchymal markers in MCF7 and A549 cancer cells that normally display epithelial traits (Fig. 2a). To this end, we ectopically expressed a DDK-tagged wild-type (WT) STAMBPL1 in these cells and found elevated vimentin as well as suppressed E-cadherin protein levels compared to the control vector (Fig. 3a), suggesting that augmented expression of STAMBPL1 could facilitate EMT. Since molecular EMT can differ from morphological and functional EMT, we selected A549 cells expressing the WT STAMBPL1 to investigate cell morphology for up to 10 days. Compared to the control cells (Fig. 3b, left panel), STAMBPL1 expression induced time-dependent morphological changes resembling a slight elongated mesenchymal cell-like shape (Fig. 3b, right panel). In addition, A549 cells showed enhanced migratory capability upon STAMBPL1 expression (Fig. 3c).

Since STAMBPL1 is linked to mesenchymal traits, it suggests that it may be of prognostic value. To examine this, we conducted a Kaplan–Meier survival analysis of 492 patients with lung cancer expressing STAMBPL1. Patients were categorised into low and high expressing groups by performance of all possible cut-off values between the upper and lower quantiles, with the best-performing threshold cut off presented (Fig. 3d). This analysis showed that expression of STAMBPL1 is significantly (*p* < 0.01) associated with poor prognosis. This is consistent with recent studies that found high amounts of STAMBPL1 protein in prostate cancer PC3 (derived from bone metastasis) and DU145 cell lines (derived from brain metastasis). In addition, several types of carcinomas have been found to acquire tumour-initiating capability after induction of EMT program,^16^ including colorectal,^12,30^ pancreatic^31^ and renal,^32^ all in which STAMBPL1 protein is shown to be expressed at high levels according to the Human Protein Atlas.^33^ To examine whether a differential STAMBPL1 expression could be observed in patient biopsies on the protein level, we undertook an immunohistochemical approach comparing STAMBPL1 expression with the EMT markers, E-cadherin and vimentin, in primary and metastatic tumour from the same patient with lung adenocarcinoma. This analysis showed higher STAMBPL1 expression in the metastatic lesion (Fig. 3e), along with more vimentin and reduced E-cadherin protein staining, further supporting a role of STAMBPL1 in an aggressive cancer phenotype.

We next assessed if STAMBPL1 deletion-mediated EMT effects could be rescued by re-expression of the WT, but not with a catalytically dead mutant of STAMBPL1. To this end, we generated a mutant STAMBPL1 protein. Since the enzymatic DUB activity of STAMBPL1 is dependent on two Zn^2+^-coordinating residues, E292 and D360, in the JAMM core^34^ (Fig. 3f), we replaced the aspartic acid at position 360 with alanine (D360A) in the active site of STAMBPL1 by site-directed mutagenesis. Next, by ectopically expressing the WT or mutant in NCI-H838 STAMBPL1 KO cells, we analysed if the observed gain-of-expression changes on the epithelial marker E-cadherin and ZO-1 transcript levels in the KO cells (as shown on Fig. 2f) could be reversed. While both the WT and mutant STAMBPL1 proteins were expressed at comparable levels (Fig. 3g and Supplementary Fig. S2b), re-introduction of the WT, but not the mutant, partially yet significantly suppressed E-cadherin and ZO-1 gain of expression (Fig. 3g). Additionally, we found that reintroduction of the WT significantly rescued the KO-mediated migration inhibition of NCI-H838 cells (Fig. 3h). However, the effect of the catalytically dead mutant in reversing migration inhibition was less than its influence of the molecular EMT changes as described above.

### STAMBPL1 affects the stability of the EMT-inducing TF SNAI1

**Fig. 4:**
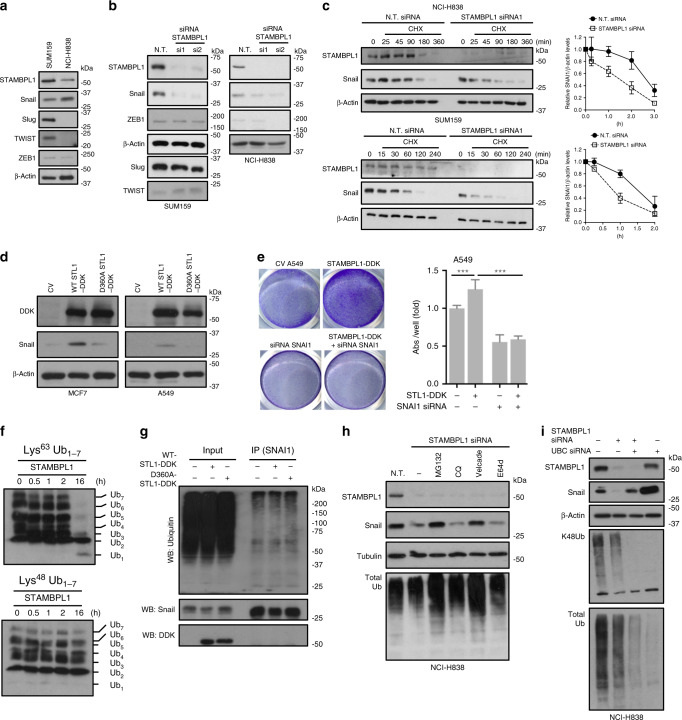
STAMBPL1 affects the stability of the EMT-TF SNAI1. **a** Western blot analyses of STAMBPL1 and indicated EMT-TFs in SUM159 and NCI-H838 cells, and **b** following transfection with N.T. or STAMBPL1 siRNAs for 48 h. **c** Cycloheximide (CHX) chase analysis of Snail protein in SUM159 and NCI-H838 cells transfected as in **b** and treated for the indicated times. Quantification of SNAI1/ACTN is shown (*n* = 3). **d** Western blots showing the effect of exogenous expression of DDK-tagged WT or mutant STAMBPL1 (D360A-DDK) for 48 h on Snail protein level. **e** Transwell migration assay of A549 cells expression a control or WT STAMBPL1-DDK plasmid, in the presence of absence of SNAI1 siRNA. Quantification is presented as fold over control (*n* = 3). **f** Lys^63^ and Lys^48^ DUB activity assays assessed by in vitro cleavage of polyubiquitin chains in the presence of recombinant STAMBPL1 protein for the indicated time points. **g** In vitro Snail ubiquitination level in HEK293T cells co-transfected with the indicated plasmids and treated with MG132 (10 μM) for 5 h before being harvested. Extracts were immunoprecipitated with anti-Snail antibody, followed by Western blotting with anti-ubiquitin and anti-DDK antibodies. **h** Western blot of Snail in the presence or absence of proteasome (MG132, Velcade) or lysosome (CQ, E64d) inhibitors in NCI-H838 cells transfected with N.T. or STAMBPL1 siRNA. Total ubiquitin accumulation indicates inhibition of the ubiquitin-proteasome pathway. **i** NCI-H838 cells depleted of *STAMBPL1* or *UbC* alone or in combination for 48 h, and analysed for Snail protein level by Western blotting. Lys^48^ polyubiquitination and overall ubiquitin decline indicate the *UbC* siRNA knockdown efficiency. β-Actin or Tubulin was used as a loading control for Western blot analysis. Data are presented as mean ± SD (*n* = 3). Statistical significance is shown over the control ***p < 0.0001 (Student’s *t* test).

For mechanistic investigation of STAMBPL1 function in the EMT program, we examined whether any of the master EMT-inducing TFs, Snail (SNAI1), Slug (SNAI2), TWIST and/or ZEB1, may be under the control of STAMBPL1. Thus, we first analysed their endogenous expression profiles in SUM159 and NCI-H838 cells (Fig. 4a). Both cells expressed STAMBPL1 along with Snail, while detectable protein levels of Slug and TWIST was only observed in SUM159 cells, and ZEB1 was expressed at low levels in both cell lines (Fig. 4a). By genetically depleting STAMBPL1 in these cells, we observed a primary effect on Snail protein levels (Fig. 4b). Less or no effect was observed on the other TFs (Fig. 4b). Cycloheximide chase experiments further revealed that STAMBPL1 depletion was associated with a shortening of Snail protein half-life (Fig. 4c), without affecting the *SNAI1* mRNA expression (Suppementary Fig. S3a). This suggests a role for STAMPBL1 in regulating Snail protein stability. Since STAMBPL1 has been shown to be an indirect activator of nuclear factor-κB) (NF-κB) signalling,^35^ and given that this pathway promotes Snail stabilisation through the synthesis of COP9 signalosome 2 protein (CSN2),^36^ we assessed if CSN2 is relevant for STAMBPL1 effect on Snail. We analysed the expression of CSN2 by qPCR and Western blotting in MCF7 and A549 cell lines subsequent to the expression of either WT or D360A mutant STAMBPL1. However, no substantial changes were observed either on the mRNA or protein levels of CSN2 in both cell lines, or on the phospho-IκBα levels, which is indicative of NF-κB activity (Supplementary Fig. S3b, c). Yet, despite the comparable expression level of ectopic WT or mutant STAMBPL1 in A549 and MCF7 cells (Fig. 4d and Supplementary Fig. S3b), only the WT STAMBPL1 resulted in a significant increase in Snail protein, compared to the control or the mutant (Fig. 4d). These data confirm that the DUB activity of STAMBPL1 contributes to the stability of Snail, likely through deubiquitination mechanisms. Further, when Snail was depleted, the effect of ectopic expression of the WT STAMBPL1 on cell migration diminishes (Fig. 4e). STAMBPL1 is, however, a DUB characterised to have a specificity for lysine-63 (K63)-ubiquitin chain linkage that is not commonly considered to target proteins to the proteasome.^4^ This was demonstrated by a matrix-assisted laser desorption/ionisation-time of flight (MALDI-TOF) DUB assay, in which systematic assessment of recombinant human DUBs against all possible ubiquitin chain linkages, highlighted STAMBPL1 K63 linkage specificity, even though at higher enzyme concentrations, STAMBPL1 show some, albeit very low, K48, K33 and K11 activities.^37^ We assessed the time-dependent ability of STAMBPL1 to cleave Lys^48^- or Lys^63^-linked ubiquitin chains. Our results were consistent with previous literature, showing that STAMBPL1 primarily cleaves Lys^63^-linked ubiquitin chains as observed by a concurrent decrease in polyubiquitin chains and appearance of Ub_1_ (Fig. 4f). Consistently, in in vitro ubiquitination assay, using HA-tagged ubiquitin in HEK-293T cells transfected with exogenously expressed Snail with WT or mutant STAMBPL1, we observed that neither the WT or the mutant resulted in direct Snail deubiquitination (Fig. 4g), indicating that the effect of STAMBPL1 on Snail is likely to be secondary, and that the Snail protein may become unstable as cells revert to an epithelial phenotype in STAMBPL1 deficiency. K63 ubiquitination can facilitate subsequent assembly of K48 leading to proteasome-mediated degradation,^38^ which could explain our data showing the effect of DUB activity of STAMBPL1 on Snail without direct deubiquitination. Thus, we assessed if STAMBPL1 knockdown-mediated Snail reduction could be hindered by inhibiting the proteasome using either MG132 or Velcade (Bortezomib) and found that the proteasome inhibitors effectively blocked Snail degradation, while lysosomal inhibitors, including chloroquine (CQ) and E64d, displayed less or no effect (Fig. 4h and Supplementary Fig. S3d). This predicts that inhibition of K48 polyubiquitination could similarly block Snail degradation. By downregulating the level of Ubiquitin using RNA interference-mediated knockdown of *Ubb* and *Ubc*, we found that knockdown of *UbC* that instructs tandem units of ubiquitin with nine repeats resulted in more efficient attenuation of both K48 and total and mono-Ub levels without affecting STAMBPL1 levels (Supplementary Fig. S3e) and that downregulation of UbC effectively hindered STAMBPL1-mediated Snail protein reduction (Fig. 4i). Of note, we also observed that basal Snail levels were further accumulated upon *UbC* gene knockdown compared to the control samples. Combined, these data clearly demonstrate that blocking the proteasomal degradation or inhibiting poly-ubiquitination both attenuates the effect of STAMBPL1 on Snail, suggesting a role for STAMBPL1 to control Snail stability that enables Snail to avoid proteasome-dependent degradation. This is further supported by reintroduction of Snail1 that blunts the suppressive effects on migration by STAMBPL1 deficiency (Supplementary Fig. S2c).

Since our data clearly indicate that STAMBPL1 affects Snail stability via the proteasome, we assessed if the levels of the main ubiquitin ligases or DUBs involved in the regulation of Snail half-life are modulated by STAMBPL1. We performed both qPCR and Western blotting to determine the transcript and protein expression levels of bTRCP1, Fbxl14, Dub3 (Usp17L2) and Usp27X in SUM159 and NCI-H838 cells depleted of STAMBPL1, as well as in STAMBPL1 KO NCI-H838 cells, compared to N.T. siRNA or WT control cells, respectively. This analysis revealed no major differences in the expression levels of any of these proteins (Supplementary Fig. S3f–h), indicating that STAMBPL1 acts independently of ubiquitin ligases or DUBs previously implicated in the control of Snail.

### Prevalence of a STAMBPL1-SNAI1 co-signature across a spectrum of tumour types

**Fig. 5:**
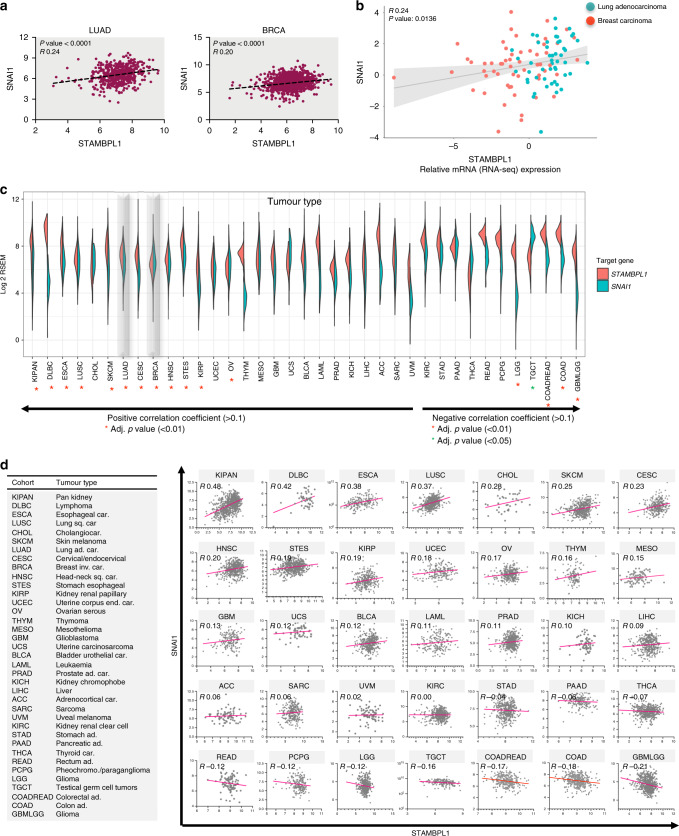
Co-signature profile of STAMBPL1 and SNAI1 across multiple tumour types. **a** Linear regression analysis of *STAMBPL1* and *SNAI1* expression from TCGA cancer datasets for LUAD and BRCA. **b** Tumour genomic RNA-seq expression data from 107 human lung adenocarcinoma and breast cancer cell lines analysed for expression correlation of *STAMBPL1* with *SNAI1* using the CCLE dataset with indicated *R* and *p* values. **c** Split-violin plot showing the expression distribution and co-occurrence of *STAMBPL1* and *SNAI1* in 37 human tumour types. Positive or negative correlations are indicated as arrows, with indicated adj. *p* value significant marked with red or green stars. **d** (left) The TCGA tumour types and (right) scatterplots showing linear regression analysis correlation of *STAMBPL1* (*x*-axis) and *SNAI1* (*y*-axis) by tumour type. The COADREAD and COAD display similar plots, as COADREAD cohort has more patients and COAD is a sub-cohort from COADREAD.

To substantiate the link between STAMBPL1 and Snail, we probed their clinical relevance in LUAD and BRCA tumour samples retrieved from the TCGA data sets. This revealed a significant positive correlation between *STAMBPL1* and *SNAI1* in both tumour types, with a *p* value <0.0001 (Fig. 5a). The tumour genomic RNA-seq expression data from 107 human lung adenocarcinoma and breast ductal carcinoma lines (Fig. 5b) further confirmed a significant (*p* < 0.05) positive *STAMBPL1*-*SNAI1* expression profile.

Since Snail is frequently shown to be highly expressed in many cancer cells^39^ and due to its strong association with high tumour recurrence rates, we hypothesised that *STAMBPL1* may be co-elevated with *SNAI1* in tumour types beyond LUAD and BRCA. Hence, we analysed 35 additional human tumour types for a STAMBPL1-SNAI1 co-signature (Fig. 5c, d). The distribution of mRNA expression levels of *STAMBPL1* and *SNAI1* was analysed with split-violin plots, which shows the expression and the co-occurrence of *STAMBPL1* (red) with *SNAI1* (green) in each tumour type, including LUAD and BRCA (Fig. 5c). As a higher expression distribution does not necessary imply a positive correlation, we computed and ranked the tumour types by the correlation coefficient from high to low (left to right), with adj. *p* value significant (0.01) positive correlations indicated with red stars. Importantly, we found that 24 tumour types showed a positive correlation, out of which >4000 samples in 12 TCGA tumours displayed a significant STAMBPL1-SNAI1 co-expression profile (Fig. 5d). In the remaining 10 tumour types, 9 showed a negative correlation coefficient score, out of which 4, including LGG, COADREAD, COAD and GBMLGG, displayed statistical significance with adj. *p* value <0.01, and 1 type TGCT with adj. *p* value <0.05. Altogether, these data highlight the prevalence of the identified STAMBPL1-SNAI1 association across an extensive number of tumours types.

### STAMPBL1 is expressed during different EMT-promoting conditions

So far, our data highlight STAMBPL1 as a significant contributor of the EMT process. Yet, it remains elusive as to how STAMBPL1 itself may be regulated during EMT. Due to the multi-dimensional landscape of cancer cells to undergo EMT, we explore during which EMT-promoting condition(s) STAMBPL1 may be involved in. We employed the tumour growth factor-*β* (TGF-*β*) or pharmacologically inhibited the activity of glycogen synthase kinase-3 (GSK-3) with LiCl, and induced ROS by Paraquat treatment in A549 cells, as commonly used means to trigger EMT, and confirmed that these treatments promoted EMT indicated by a loss of E-cadherin and increased vimentin (Supplementary Fig. S4a). LiCl induced a significant Snail accumulation, albeit to a lower degree than TGF-*β*, thus instead of LiCl that only partially inhibit GSK-3α/β activity,^40^ we tested a more specific molecule inhibitor of GSK-3α/β (CAS 667463-62-9), to compare the effect of GSK-3α/β inhibition or TGF-*β* stimulation on STAMBPL1 expression in A549 and MCF10A cells. A marked increase in STAMBPL1 levels were shown upon inhibition of GSK-3α/β in both cell lines, while its expression was not induced upon TGF-*β* treatment (Supplementary Fig. S4b). Thus, we further examined the effect of constraining GSK-3α/β activity in MCF7 cells, known to be less sensitive to TGF-*β*, and found that STAMBPL1 levels were similarly upregulated in MCF7 cells upon GSK-3α/β inhibition with simultaneous significant induction of Snail protein level (Supplementary Fig. S4c, d). Furthermore, silencing of STAMBPL1 prior to treatment with GSK3α/β inhibitor for 24 h blunted its effect on *VIM* mRNA elevation (Supplementary Fig. S4e). These data suggest a role for STAMBPL1 in EMT-promoting mechanisms that involve inhibition of GSK-3β activity and indicate a difference in STAMPBL1 expression during various EMT-promoting conditions.

### Mutant p53 induces STAMPBL1 expression in MCF-10A cells

**Fig. 6:**
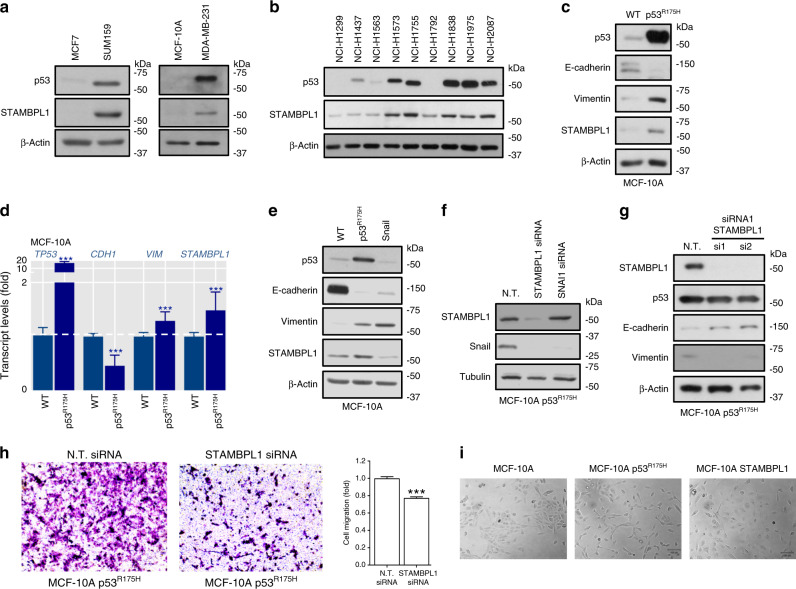
STAMBPL1 expression is enhanced by oncogenic mutant p53. **a**, **b** Western blots showing the expression of p53 accumulation and STAMBPL1 protein level in indicated breast and lung cancer cell lines. **c**, **d** Protein expression (Western blots) and relative mRNA expression of *TP53*, *CHD1*, *VIM* and *STAMBPL1* in parental or stable mutant p53^R175H^-expressing MCF-10A cells. **e** Western blots of indicated EMT marker proteins in WT, mutant p53^R175H^ and Snail stable expressing MCF-10A cells. **f** Western blots of STAMBPL1 and Snail in mutant p53^R175H^ MCF-10A cells transfected for 48 h with N.T., STAMBPL1 or Snail siRNAs. **g** Western blots of p53 and indicated EMT marker proteins in mutant p53^R175H^ MCF-10A cells transfected for 48 h with N.T. or STAMBPL1 siRNAs. **h** Transwell migration in p53^R175H^ MCF-10A cells transfected for 48 h with N.T. or siRNA targeting STAMBPL1. Quantification is presented in normalised bar graphs (*n* = 3). **i** Morphology of control, p53^R175H^ and WT STAMBPL1-DDK expressing MCF-10A (normal light microscopy, magnification ×20), scale bar, 100 μm. Data are presented as mean ± SD (*n* = 3). Statistical significance is shown over the control. *p < 0.05, **p < 0.001 and ***p < 0.0001 (Student’s *t* test). β-Actin was used to ensure equal loading for all Western blot analysis.

GSK-3 activity is involved in many signalling pathways and known to be under the regulation of the tumour suppressor protein p53.^41^ Bidirectional inhibition of GSK-3 activity is shown to increase the abundance of p53.^42^ While p53 mediates changes in gene expression that inhibit EMT,^43^ in many human cancers, the existence of mutant p53 proteins display oncogenic gain-of-function (GOF) activities with a robust capacity to promote EMT.^26,44^ We speculated that STAMBPL1 might be connected to oncogenic signalling pathways connected to GSK-3β activity, which prompted us to examine the relationship between mutant p53 and STAMBPL1. Accordingly, beyond the considerably low expression of STAMBPL1 in the p53^WT^ MCF7 cells in comparison to the p53^R158InF^ SUM159 cells, we observed that the p53^WT^ MCF-10A cells similarly displayed lower STAMBPL1 levels compared to the MDA*-*MB*-*231 cells exhibiting p53^R280K^ mutant (Fig. 6a). Beyond breast cancer, to further strengthen this association, we explored if endogenous mutant p53 levels correlated with STAMBPL1 expression in a panel of human NSCLC cell lines (Fig. 6b). A marked higher expression of STAMBPL1 was observed in all cell lines with a pronounced mutant p53 protein expression (Fig. 6b), indicating a link between STAMBPL1 and mutant p53 expression in malignant cells. Notably, NCI-H1299 and NCI-H1792 cells, exhibiting *TP53* mutations causing a null expression, showed less STAMBPL1 expression, likely to indicate that the effect on STAMBPL1 is not as a result of loss of function of WT cells, but rather a GOF of mutant p53.

To functionally investigate whether STAMBPL1 is under the regulation of mutant p53, we employed the parental WT and p53^R175H-^expressing MCF-10A cells, in which EMT is triggered by the mutant GOF.^45,46^ Since the stable expression of p53^R175H^ in MCF-10A was created in a null p53 background,^26^ the dominant-negative activity of the mutant protein on WT p53 could be excluded. Using these cells, we first reverified that p53^R175H^ indeed represented an EMT model by analysing the mRNA and protein changes in vimentin and E-cadherin levels (Fig. 6c, d). Concomitantly, we observed that STAMBPL1 expression was elevated in cells with mutant p53-elicited EMT (Fig. 6c). Similarly, *STAMBPL1* mRNA was enhanced in p53^R175H^ cells, compared to the parental MCF-10A line (Fig. 6d), indicating a role for mutant p53 in stimulating STAMBPL1 expression.

Consequently, we explored the possibility of transcriptional regulation of STAMBPL1 by mutant p53. To this end, we first performed a p53-binding motif search by using EPD (Eukaryotic Promoter Database) on STAMBPL1 from −12,000 to +1000 bp relative to TSS (transcription start site) with a cut-off *p* value of 0.001 and found four nonconsensus DNA-binding motif sites predicted for p53 in the region (−2000 to +1 bp) on STAMBPL1, and one putative p53 DNA-binding consensus motif within the distal (−10,609) promoter region (Supplementary Fig. S5a). As a complement to this search, we inquired information from the p53 Bing Loci Database (http://cosbi4.ee.ncku.edu.tw/p53BLD/converter/Home), which collected 13 publicly available p53 ChIP-seq datasets from various cell lines and p53 mutant variant. Retrieved data showed that only mutant p53^R248W^ possess a peak location on STAMBPL1 in Li-Fraumeni fibroblast (MDA-H087), while WT and other p53 mutants showed no binding with *STAMBPL1* gene across all the 13 datasets.

Next, by using primers to specifically amplify the six predicted sites, including the peak location possessed by mutant p53^R248W^ retrieved in p53 Bing Loci Database (Supplementary Fig. S5a), we performed ChIP-qPCR experiments on p53^WT^, untreated or MG132 treated to increase WT expression, as well as in p53 ^R175H^ MCF-10A cells (Supplementary Fig. S5b). As positive controls, p53 occupancy on *MDM2* and *CDKN1A*, two well-known p53 target genes, was determined. While ChIP assay revealed p53 enrichment on MDM2 and CDKN1A promoters in p53^WT^-expressing cells (up to ~4% and ~10% of input), no significant (>1%) occupancy on any of the six predicted was detected for either WT or p53 ^R175H^ (Supplementary Fig. S5c), indicating that the expression of *STAMBPL1* gene is not directly regulated by p53^R175H^.

Next, to examine if STAMBPL1 expression is due to an indirect consequence of EMT-induced by mutant p53^R175H^, we compared the effect of Snail-induced EMT on STAMBPL1 in MCF-10A cells. While these cells effectively underwent EMT, STAMBPL1 levels were not increased (Fig. 6e). Moreover, while knockdown of STAMBPL1 caused a significant decrease in Snail levels, STAMBPL1 levels were unaffected upon Snail depletion in mutant p53^R175H^ MCF-10A cells (Fig. 6f). Further, when we assessed the effect of STAMBPL1 knockdown in mutant p53^R175H^ MCF-10A cells, we observed re-expression of E-cadherin and vimentin reduction (Fig. 6g), whereas STAMBPL1 knockdown did not affect mutant p53. These data suggest a functional role for STAMBPL1 in mutant p53-mediated EMT in MCF-10A cells.

Conclusively, by performing both transwell and wound scratch assays, we observed that STAMBPL1 depletion showed significant effects on the migratory capacity of p53^R175H^ MCF-10A cells (Fig. 6h and Supplementary Fig. S5d), whereas ectopic expression of STAMBPL1 induced a more spread and stretched morphology on MCF-10A cells similarly to the changes elicited by stable mutant p53^R175H^ expression (Fig. 6i). Collectively, these findings uncover a novel concept of mutant p53^R175H^ oncogenic regulation of a DUB that impacts EMT. Yet, additional regulatory mechanism may control STAMBPL1 expression in other contexts and cells.

## Discussion

In this study, by undertaking systematic correlation screenings of human DUBs with mesenchymal markers in primary biopsies of LUAD and BRCA, we uncovered a unique DUB, the zinc metalloprotease STAMBPL1, as an important contributor of the EMT program. STAMBPL1 was initially discovered as an AMSH (associated molecule with the SH3 domain of STAM) family protein,^45^ able to enhance interleukin-2-mediated induction of the oncogene *c-myc*. Yet, due to the uncharacterised direct STAMBPL1 substrates, its cellular function has remained unclear. More recently, STAMBPL1 has been implicated as an endosomally associated protein found to be an indirect activator of NF-κB signalling,^35^ while its depletion is implicated to induce apoptosis.^46^ However, no studies have previously linked STAMBPL1 to EMT regulation. Here, by assessing the role of STAMBPL1 in EMT using three approaches: analysis of gene expression data from human tumours and its relevance with EMT markers in patient material, phenotypical functional analyses in cell lines models of EMT and its potential regulation during the EMT program, we present substantial amount of data underscoring the significant correlation between mesenchymal phenotype with STAMBPL1 and propose STAMBPL1 as a predictive marker for EMT.

STAMBPL1 does not belong to the classical proteasomal DUBs due to its linkage specificity, yet its depletion is shown to affect the stability of proteins indirectly by targeting them to either proteasomal ((HTLV-1) Tax oncoprotein) or lysosomal degradation (XIAP).^35,46^ In accordance with this role, we show that the STAMBPL1 is involved in indirect proteasomal regulation of the TF Snail. Our data further suggest that STAMBPL1 may primarily affect Snail and not the other EMT-TFs,^47^ thus it may be specific for this member of EMT-TFs. While our study does not address the mechanistic details on this regulation, we uncover a substantial STAMBPL1-SNAI1 link across multiple human tumours. However, we do not exclude that STAMBPL1 might have other direct or indirect targets that may influence EMT beyond Snail, and thus signalling events that can act pleiotropically to choreograph with each other to promote the complex EMT program.

While Snail plays a crucial role in triggering EMT upon multiple stimuli, a correlation between STAMBPL1 expression and SNAIL increase was not observed in all EMT conditions, triggered by different means. The fact that different cancer cells are differently susceptible to various EMT stimuli, in addition to that Snail is regulated by various E3 ligases in response to diverse stimuli, as a part of the different path cancer cells can take to undergo EMT in the multi-dimensional landscape, strongly suggests the possibility that distinct DUBs may be equally important in Snail regulation to different EMT stimuli. In fact, DUBs involved in Snail regulation so far are shown to be induced differently, while USP27x is induced by TGF-*β*;^48^ DUB3/USP17L2 seems to play a role in CDK4/6-mediated activation of EMT,^49^ whereas OTUB1 is under the transcriptional regulation of oestrogen-related receptor alpha,^50,51^ and USP37 regulation is induced during EMT via the stimulation of the hedgehog signalling pathway.^52^ Consistent with the effect of GSK-3 inhibition to stimulate the transcription of Snail,^53,54^ in this study, we show that GSK-3α/β inhibition can induce a significant STAMBPL1 expression in all cell lines tested, signifying that this stimulation is able to trigger similar STAMBPL1 molecular response in several cell and cancer types and suggest that GSK-3α/β inhibition may converge upon common signalling element in multiple carcinomas to activate STAMBPL1. Hence, the uncovering of STAMBPL1 and its regulatory mechanism constitute an important advancement to our understanding of how a DUB can contribute to the EMT program.

Furthermore, our data propose a novel regulatory mechanism of DUBs by oncogenes, as we identify a functional role for mutant p53 in STAMBPL1 expression. Until now, despite the extensive literature on DUBs, whether oncoproteins whose upregulation is required for cancer enhancement could affect DUBs remained unclear, which may in part likely reflect variation in DUB abundances in different tumour types. While our study highlights and connect three key signalling pathways: mutant p53, EMT and DUBs, showing that STAMBPL1 is transcriptionally elevated in mutant p53-expressing cells, STAMBPL1 seems not to be under a direct regulation by p53^R175H^. However, out of the known TFs that help recruit mutant p53 to the chromatin to upregulate genes,^55^ we found that E2F2, E2F6, MAFF and SP1 can bind to the core promoter region of STAMBPL1 by their motif search using Eukaryotic promoter database. Thus, the mechanism by which mutant p53 might activate STAMBPL1 is therefore likely through its interaction with these TFs, which possess responsive to mutant p53.^55–57^ Although future studies are needed to delineate whether other DUBs are involved in mutant p53-induced EMT or to other mutant p53-mediated oncogenic pro-survival benefits (ref. ^58,59^), our findings suggest an involvement of mutant p53 in driving the expression of STAMBPL1 as a novel pro-EMT GOF, providing further rationale to investigate DUB expression by other oncoproteins.

Conclusively, by defining a role of STAMBPL1 in EMT and as a potential therapeutic target in multiple human carcinomas, we propose that targeting of STAMBPL1 may provide an alternative approach for treatment of cancer progression by exhausting the EMT potential of cancer cells. In addition, a mutant p53-dependent mechanism that controls DUBs in EMT may be instrumental in developing specific inhibitors and/or activators, serving as future DUB-targeting strategies and therapeutic modalities against the growing number of pathologies associated with mutant p53.

## Supplementary information

Supplementary Material

## Data Availability

All original WB are available as Supplementary figures. All other data are available within the article and its supplementary information files.
