# Supplementary material for: Systematic analysis reveals a functional role for STAMBPL1 in the epithelial–mesenchymal transition process across multiple carcinomas
Source: Br J Cancer. 2020 Jul 8;123(7):1164–77. doi: 10.1038/s41416-020-0972-x (PMC7524718; doi:10.1038/s41416-020-0972-x)

**Supplementary information Material and Methods**

**Human cancer cell lines and treatment conditions**

The human NSCLC: NCI-H838, NCI-H1299, NCI-H1437, NCI-H1563, NCI-H1573 NCI-H1755, NCI-H1792, NCI-H1838, NCI-H1975, NCI-H2087 and A549 cell lines were cultured as described previously,[25] in RPMI (Sigma-Aldrich) medium. The human breast cancer MCF-7 and MDA-MB-231 cell lines was cultured in DMEM (Gibco) medium as described before.[58,59] The RPMI and DMEM medium were supplemented with 10% (v/v) heat-inactivated fetal bovine serum (FBS) (Gibco), 100 U/ml penicillin and 100 U/ml streptomycin (Sigma-Aldrich) and 1% (w/v) glutamine (Sigma-Aldrich). For MCF7 cells 10 μg/ml insulin (Sigma-Aldrich) was further added to the medium. The human breast cancer cell line, SUM159, was cultured in Ham's F12 medium (Lonza) supplemented with 5% (v/v) heat-inactivated FBS (Gibco), 100 U/ml penicillin and 100 U/ml streptomycin (Sigma-Aldrich), 5 mg/mL insulin and 1 mg/mL hydrocortisone. The human normal epithelial breast MCF-10A cells were cultured in DMEM/F12 (Gibco) supplemented with 5% (v/v) horse serum (Gibco), 20 ng/ml Epidermal Growth Factor (EGF) (Peprotech), 0.5 mg/ml Hydrocortisone (Sigma-Aldrich), 100 ng/ml Cholera Toxin (Sigma-Aldrich), 10 μg/ml Insulin (Sigma-Aldrich) and 100 U/ml penicillin and 100 U/ml streptomycin (Sigma-Aldrich).

All cells were grown in a humidified 5% $CO_2$ atmosphere at 37°C and maintained in a logarithmic growth phase. Cell proliferation was assessed by counting the number of cells over time. Equal cell number/well was seeded in triplicates at day one and cell numbers were monitored up to 3 days, every 24 h.

The following reagents were used to treat cells at the indicated concentrations: 5 ng/ml TGF-β1 (100-21C) (PeproTech), 200 nM PQ (Paraquat) (Sigma-Aldrich), 15 mM LiCl (Lithium chloride) (Sigma-Aldrich), 10 μM GSK-3 Inhibitor IX (CAS 667463-62-9 Calbiochem), 2 μM MG132 (Sigma-Aldrich), 1 μM Velcade (B-1408 Bortezomib) (LC Laboratories), 50 μM CQ

(Cloroquin) (Sigma-Aldrich, C6628), or 5 µM E64D (Sigma-Aldrich, E8640). For Cycloheximide chase assay, cells were seeded on a 12-well plate and treated with 10 µM CHX (Sigma-Aldrich) for up to 4 h including 5 different time points. After treatment, cells were harvested and lysed before the resulting samples were run for Western blotting.

**RNA extraction and cDNA synthesis**

RNA was isolated with the use of the PureLink™ RNA Mini Kit (Thermo Fisher Scientific) and DNA was removed using PureLink™ DNase Set (Thermo Fisher Scientific). RNA (1 µg) was synthesized to cDNA with the IScript cDNA synthesis kit (Bio-Rad) according to the manufacturer's instructions. Quantitative PCR (qPCR) was performed with 200 ng of the cDNA sample using Maxima qPCR SYBR green master mix (Thermo Fisher Scientific) and amplified with the 7500 real-time PCR system (Applied Biosystems). The ΔΔCT method was applied to determine the relative mRNAs expression. *β*-actin or Tubulin was used as the reference gene. Primer sequences are stated in the supplementary information.

**Quantitative PCR analysis.** The following oligonucleotides were used:

| Gene Name | Sequenz (5'->3') |
|---|---|
| ACTN Fw | GCAAGCAGGAGTATGACGAG |
| ACTN Rv | CAAATAAAGCCATGCCAATC |
| BTRC Fw | CCAGACTCTGCTTAAACCAAGAA |
| BTRC Rv | GGGCACAATCATACTGGAAGTG |
| CDH1 Fw | CTCGACACCCGATTCAAAGT |
| CDH1 Rv | CCAGGCGTAGACCAAGAAAT |
| CDH2 Fw | CCTCCAGAGTTTACTGCCATGAC |
| CDH2 Rv | GTAGGATCTCCGCCACTGATTC |
| CLDN1 Fw | GTCTTTGACTCCTTGCTGAATCTG |
| CLDN1 Rv | CACCTCATCGTCTTCCAAGCAC |
| COPS2 Fw | AGGACTACGACCTGGAATACTC |
| COPS2 Rv | CCGCTTTTGGGTCATCTTCTTT |
| CTNNB1 Fw | CACAAGCAGAGTGCTGAAGGTG |
| CTNNB1 Rv | GATTCCTGAGAGTCCAAAGACAG |
| DUB3/USPL17L2 Fw | CACACTTCTGCCAAGGTCCTCA |
| DUB3/USPL17L2 Rv | AGAGGTCCTGTGTTCTGCTGAG |
| Fbxl14 Fw | TGCGCTCCTGTGACAACATC |
| Fbxl14 Rv | TGGGCTATGTAAGCCAGACTC |
| GRHL2 Fw | GAAAACCGAGTGCAAGTCCTA |
| GRHL2 Rv | GGGCCATGAAAACTGGTGTG |
| OVOL2 Fw | ACAGGCATTCGTCCCTACAAA |
| OVOL2 Rv | CGCTGCTTATAGGCATACTGC |

| | |
|---|---|
| SNAI1 Fw | GAGCCCAGGCAGCTATT |
| SNAI1 Rv | AGTGACAGCCATTACTCACAG |
| STAMBPL1 Fw | GAGGATGGCGTCTGTGTATTT |
| STAMBPL1 Rv | GCTGGTAATCTCGATGGTTAGG |
| TJP1 Fw | GTCCAGAATCTCGGAAAAGTGCC |
| TJP1 Rv | CTTTCAGCGCACCATACCAACC |
| TP53 Fw | GTTCCGAGAGCTGAATGAGG |
| TP53 Rv | TTATGGCGGGAGGTAGACTG |
| Tubulin Fw | TCTACCTCCCTCACTCAGCT |
| Tubulin Rv | CCAGAGTCAGGGGTGTTCAT |
| USP27X Fw | CAACCTGGGAAACAACCAAACC |
| USP27X Rv | GGCCTGGACAATGCAGTTCAT |
| VIM Fw | GATTCACTCCCTCTGGTTGATAC |
| VIM Rv | GTCATCGTGATGCTGAGAAGT |

**Site directed mutagenesis:** The following oligonucleotides were used:

| **Gene Name** | **Sequence (5'->3')** |
|---|---|
| STAMBPL1 D360A Fw | CAGCGTTGCTCTTCACACTCACTGTTCCTATCAACTCATGTTGC |
| STAMBPL1 D360A Rv | TGTGAAGAGCAACGCTGGATAAAAATGCAGTTTGAGTGGGATGTG |

**RNAi-mediated knockdown:** The following siRNA were used:

| **Horizon Discoveries:** | **Sequence** |
|---|---|
| NT (Non-targeting) siRNA | CGUAGAAUACCAAGAAUAU |
| STAMBPL1 #1: | CGUAGAAUACCAAGAAUAU |
| STAMBPL1 #2: | GCUAGAAUCGGAGCAGUUU |
| SNAI1 | AAUCGGAAGCCUAACUACA |
| **ON-Targetplus SMARTpool siRNA:** | **Cat no:** |
| UbB (UBB) | L-013382-00-0005 |
| UbC (UBC) | L-019408-00-0005 |
| **GenePharma** | **Sequence** |
| TP53 Sence (5'-3') | GACUCCAGUGGUAAUCUACTT |
| TP53 antisence (5'-3') | GUAGAUUACCACUGGAGUCTT |

**CRISPR/Cas9-mediated genome editing:**

| **STAMBPL1** | **Sequence (5'->3')** |
|---|---|
| Target sequence 1 | CCAGACCTAAAGTAACGTCG<u>TGG</u> |
| Target sequence 2 | AATATCACTAGCTTAGTCAG<u>GGG</u> |
| sgRNA sequence 1 | CCAGACCUAAAGUAACGUCG |
| sgRNA sequence 2 | AAUAUCACUAGCUUAGUCAG |
| Genotyping primer Fw | TGAAGATGCAGAGAAGCCCT |
| Genotyping primer Rv | TCACCAGGCTCACAATTCCA |

**CHIP-qPCR analysis**: The following oligonucleotides were used:

| **Oligo Name** | **Sequence (5'->3')** |
|---|---|
| MDM2 Fw | GTTCAGTGGGCAGGTTGACT |
| MDM2 Rv | CGGAACGTGTCTGAACTTGA |
| CDKN1A Fw | AGCCTTCCTCACATCCTCCT |
| CDKN1A Rv | GGAATGGTGAAAGGTGGAAA |
| 1_STL1(-10609) Fw | GGCTCATCCTAGACCTCCTC |
| 1_STL1(-10609) Rv | TCCATGAGCTGATGACCTCC |
| 2_STL1(-3046) Fw | CCAGGTGGCAAAAGCTATGT |
| 2_STL1(-3046) Rv | ACATTTCCTTCTGCAACGCC |
| 3_STL1(-2362) Fw | AGGAACATGGTCACTGTCTCC |
| 3_STL1(-2362) Rv | TCAGAGTTGACCACACTTGTAGT |
| 4_STL1(-2177) Fw | TCCGAAACCCTGTTCTACCA |
| 4_STL1(-2177) Rv | TGCAGATGAGAAAATGAGGGC |
| 5_STL1(+213) Fw | CAAAGCTCCATCTGTCGGC |
| 5_STL1(+213) Rv | GTTGCAGCGGGGTGATAC |
| 6_STL1(+715) Fw | GATAGGGAGGCAGGAAGACC |
| 6_STL1(+715) Rv | CTCTCCCAAGGTCGACATCA |

**Immunoblotting and immunoprecipitation**

Cells were lysed for 30 min on ice in RIPA lysis buffer (50 mM Tris, pH 7.5, 150 mM NaCl, 1% NP-40, 0.05% sodium deoxycholate, 0.1% SDS) supplemented with complete protease inhibitors (Roche Diagnostics). Sample protein concentration was determined using the BSA assay (Pierce). Equal amounts of protein from each sample were mixed with Laemmli loading buffer and subjected to SDS-PAGE. 10-15% *acryl amide gels were used*. Membranes were blocked for 1 h with 5% non-fat milk in PBS (+ tween) at RT and probed with the primary antibody of interest O/N at 4°C. Blots were revealed by ECL (BioRad) following staining with secondary antibodies 40 min (1:10 000 dilution) in 2.5 % non-fat milk in PBS (+ tween) at RT and washed in PBS.

**Antibodies:** Primary antibodies: *β*-actin (sc-81178), p53 (sc-126), STAMBPL1 (sc-376526), TWIST (sc-81417) Santa Cruz Biotechnology. E-cadherin (#3195), SNAI2 (#9589), SNAI1 (WB: 3879, IP: #3895), Vimentin (#5741), Claudin-1 (#13255), N-cadherin (#13116), ZEB1 (#3396), ZO-1 (D7D12) (#8193), *β*-Catenin (D10A8) (#8480), K63-linkage (D7A11) (#5621), K48-linkage (#4289), Phospho-IκBα (Ser32/36) (#9246) Cell Signaling Technology. DDK (TA50011) OriGene. Anti-Ubiquitin (BML-PW8810) Enzo Life Sciences. USP27X (PA5-71973), FBXL14 (PA-100521), BTRC (1B1D2), USP27L2 (PA5-44981), MYEOV2/COPS9 (PA5-100621) Invitrogen. Secondary antibodies: Goat Anti-Mouse IgG (H+L) and Goat Anti-Rabbit IgG (H+L) Peroxidase Conjugated (Thermo Scientific). All antibodies were diluted according to the manufacturer's recommendation.

**Immunofluorescence and Cell morphology**

Cell morphology was captured using an inverted microscope (Zeiss Vert A.1 AX10). Normal light, magnification ×20. For immunofluorescence, cells were seeded on coverslips in 24-wells

plate and fixed with 4% paraformaldehyde (Sigma-Aldrich) for 30 min at RT. Anti-Vimentin (#5741, Cell Signaling) antibody was blocked with PBS and 5% BSA (Sigma-Aldrich) for 2 hours at room temperature, diluted in PBS-0.3% Triton, and added to the coverslips and incubated overnight at 4°C. Cells were washed three times with PBS-0.3% Triton, incubated with an Alexa Fluor 488-conjugated secondary antibody (Thermo Fisher Scientific) for 30 min, and counterstained with DAPI (Thermo Fisher Scientific). Images were acquired in a confocal microscope Zeiss LSM710 (Zeiss, Germany). All images were analyzed using the ZEN lite software (Zeiss). The fluorescence intensity was determined using the ImageJ version 1.47t.

**Transwell Migration and Wound healing assay**

Cells were seeded in confluent conditions in 6-well plates before a scratch/wound was made with a 200 µL pipette tip the next day. Damaged and detached cells were cleared by washing the wells 3 times with PBS. Images were acquired right after the wound was made (control) and then at the indicated time points. The healing (rate of cell migration) was assessed using an inverted phase contrast microscope (Zeiss). Pictures were obtained using a 10x-objective and analyzed by Axio Vision 4.8 software (Zeiss).

The Transwell migration assay was carried out in 24-well transwell plates with inserts including a polycarbonate membrane containing 8.0 µm wide pores (Costar). Equal number of cells were seeded in the upper chamber in 400 µL of medium containing 2.5% FBS. The lower chamber was filled with 1 mL of adequate regular growth media. After 3–6 days, the membrane was washed 3 times with PBS to remove non-migrating cells. The membrane was then fixed in 4% paraformaldehyde for 15 min at RT before stained with 0.1% crystal violet for 15 min. Membranes were then washed five times in distilled water. Pictures of set of 3 different fields were taken using EVOS XL Core Imaging system (ThermoFisher Scientific) for each condition. Crystal violet staining was then dissolved by adding 500 µL 33% acetic acid in each insert/well.

The resulting eluent was collected, absorbance was measured at 560 nm and normalized to the control condition, as presented as Abs/well (fold).

# Supplementary Figures

Ambroise and Yu *et al.*, Supplementary Fig. S1

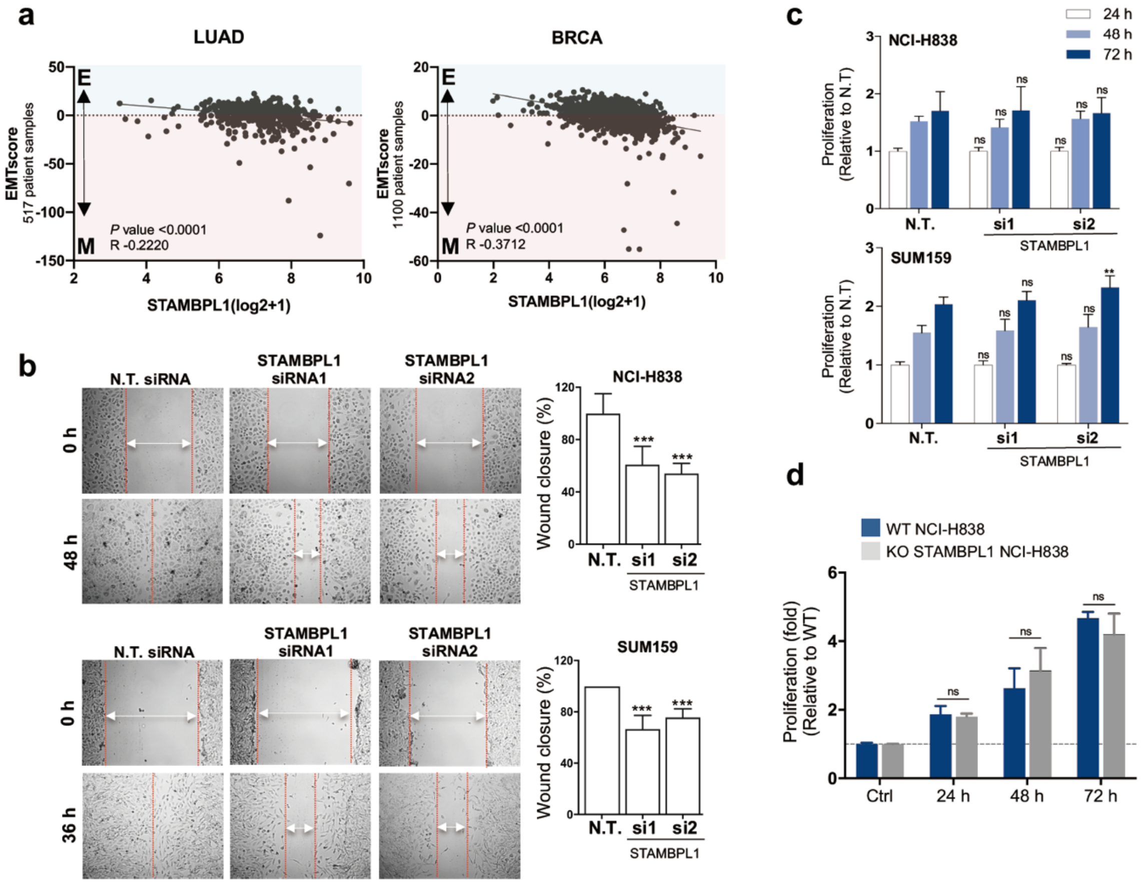


**Figure S1 Legend. a** The Pearson correlation between STAMBPL1 expression and calculated EMT scores for each LUAD and BRCA TCGA tumor samples. **b** Wound-healing assay in SUM159 and NCI-H838 cells transfected with N.T or siRNA targeting STAMBPL1 for 48 h. Quantification is presented in normalized bar graphs (n=3). **c-d** Proliferation assay over 3 days (72 h) assessed for SUM159 and NCI-H838 cells transfected with N.T or two individual siRNA targeting STAMBPL1, and in WT or STAMBPL1 KO NCI-H838 cells. Statistical significance is shown over the control. **p<0.001***p<0.0001 (Student's *t*-test). Data are presented as mean ± SD (n≥3).

Ambroise and Yu *et al.*, Supplementary Fig. S2

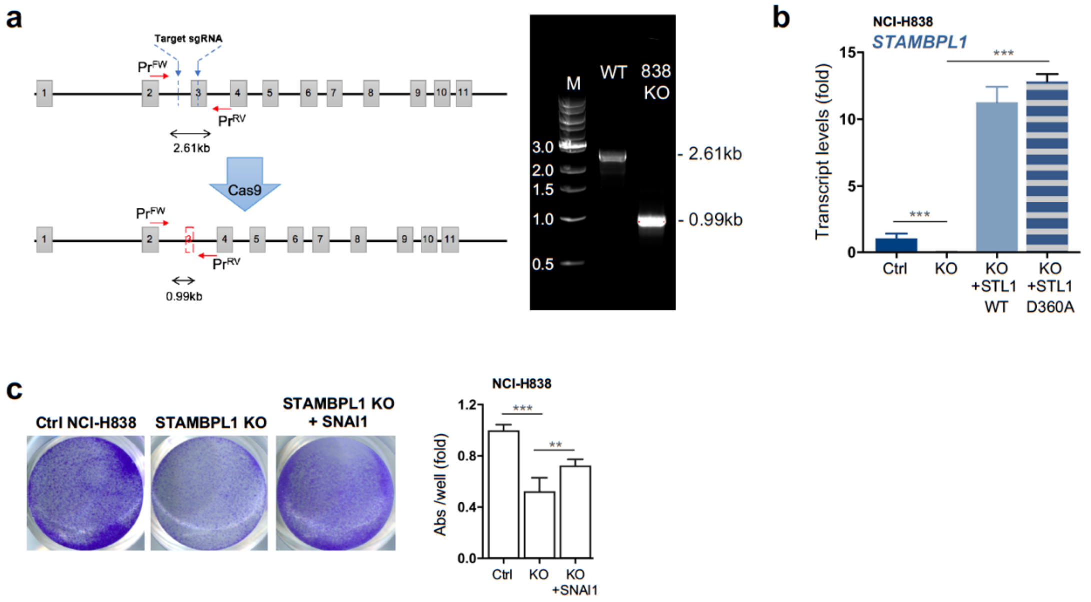


**Figure S2 Legend. a** CRISPR/Cas9-mediated genome editing approach on STAMBPL1 gene in human NCI-H838 cancer cells. The STAMBPL1 gene and the target of sgRNA/CAS9 in exon 3 of the human STAMBPL1 gene are presented with locations indicated for sequencing forward and reverse primers. Deletions introduced with the sgRNA/CAS9 ribonucleoprotein were identified by PCR using the primer set and agarose gel electrophoresis. **b** Relative mRNA (qPCR) levels of STAMBPL1 in control, STAMBPL1 KO NCI-H838 and in KO cells subsequent to ectopic expression of the WT STAMBPL1 (STL1)-DDK or catalytically inactive D360A-STAMBPL1 (STL1)-DDK. **c** Transwell migration assay in NCI-H838 control, STAMBPL1 KO cells and in KO cells subsequent to ectopic expression of SNAI1. Quantification is presented in fold over control bar graphs (n=3). Statistical significance is shown over the control. **$p$<0.001***$p$<0.0001 (Student's $t$-test).

Ambroise and Yu *et al.*, Supplementary Fig. S3

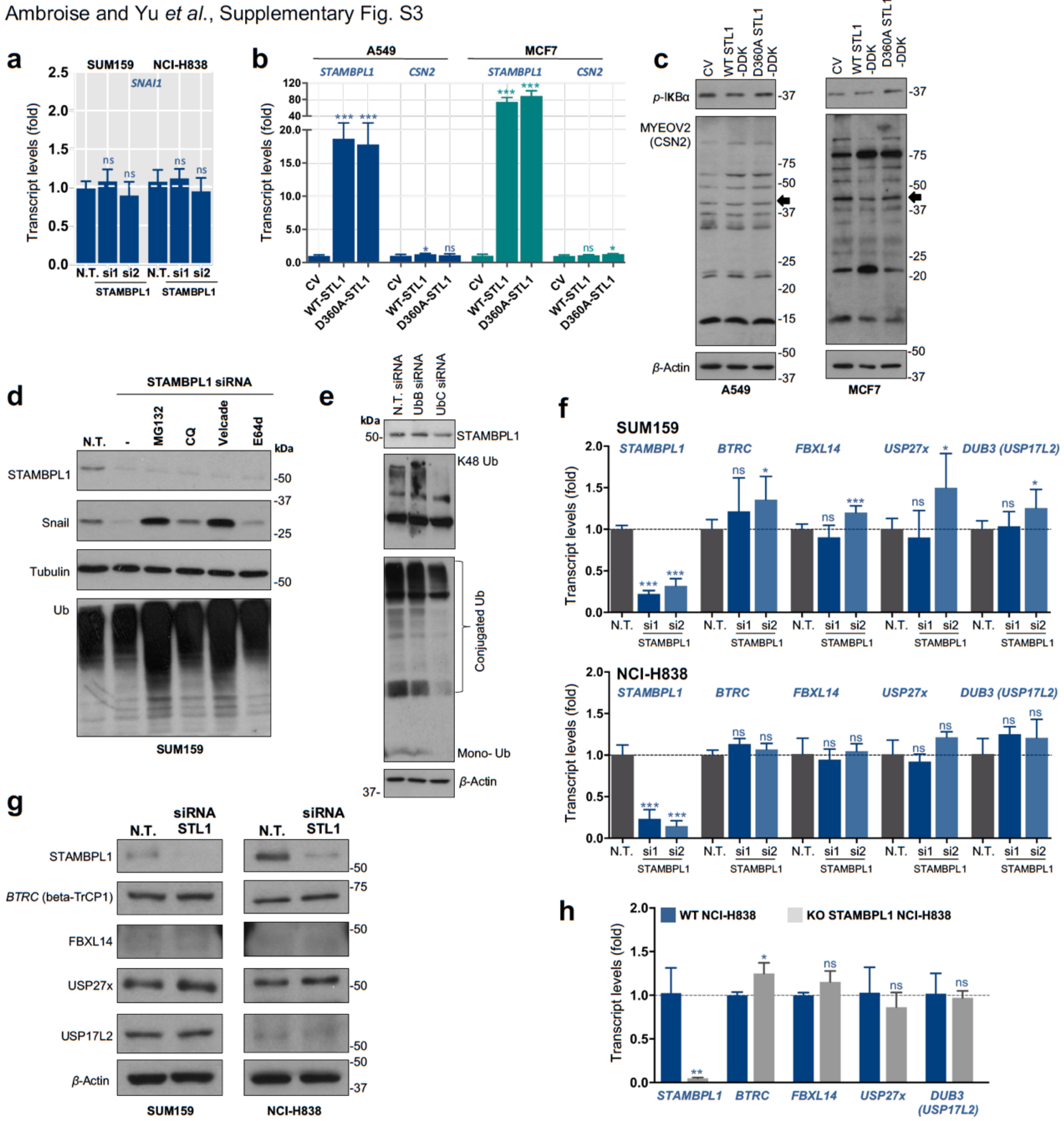


**Figure S3 legend. a** Relative mRNA levels of SNAI1 in SUM159 and NCI-H838 cells transfected for 48 h with N.T or two individual siRNA targeting STAMBPL1. **b** Relative mRNA levels of STAMBPL1 and COP9 signalosome 2 protein (CSN2) in A549 and MCF7 cells with ectopic expression of WT or D360A mutant STAMBPL1. **c** Western blots showing the levels of *p*-IKBα and CSN2 (MYEOV2) in cells treated as in b. **d** Western blot of Snail in the presence or absence of proteasome (MG132, Velcade) or lysosome (CQ, E64d) inhibitors in NCI-H838 cells transfected with N.T. or STAMBPL1 siRNA. Total ubiquitin accumulation indicates inhibition of the *ubiquitin*-proteasome pathway. **e** The effect of siRNA mediated targeting of *UbB* or *UbC* for 48 h on STAMBPL1, Lys48 polyubiquitination, total (conjugated and mono)-ubiquitin levels. **f-g** Relative mRNA expression and Western blots of of *STAMBPL1*, *BTRC*, *FBXL14*, *USP27x* and *DUB3* (*USP17L2*) in SUM159 and NCI-H838 cells transfected with N.T or siRNA targeting *STAMBPL1* for 48 h. **h** Relative mRNA expression of indicated genes as in f in STAMBPL1 WT or KO NCI-H383 cells. *β*-Actin was used as a loading control for Western blot analysis. Data are presented as mean ± SD (n=3). Statistical significance is shown over the control. *p<0.05, **p<0.001, ***p<0.0001 (Student's *t*-test).

Ambroise and Yu *et al.*, Supplementary Fig. S4

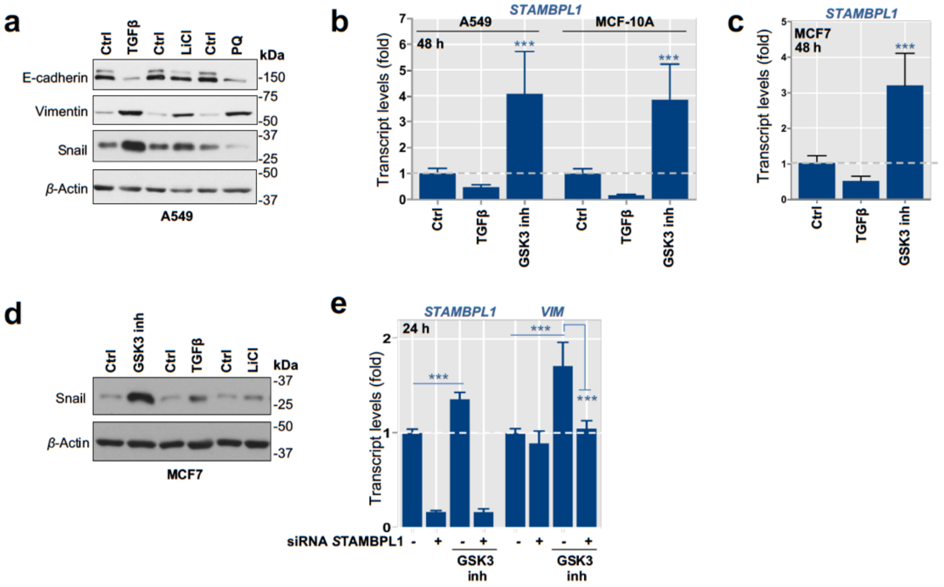


**Figure S4 legend. a** Immunoblots showing the effect of TGF-*β*, LiCl, or PQ (Paraquat) on E-Cadherin, vimentin and Snail in A549 cells treated for 48 h. **b-c** Relative mRNA expression of *STAMBPL1* in control, TGF-*β* or CAS 667463-62-9 (GSK3 inhibitor) treated A549, MCF-10A or MCF7 cells for 48 h. **d** Western blot analysis of Snail in MCF7 cells treated as indicated. **e** Relative mRNA expression of *VIM* and *STAMBPL1* in A549 cells transfected with N.T or siRNA targeting *STAMBPL1* for 48 h prior to GSK3 inhibition for 24 h. *β*-Actin was used as a loading control for Western blot analysis. Data are presented as mean ± SD (n=3). Statistical significance is shown over the control. ***p<0.0001 (Student's *t*-test).

Ambroise and Yu *et al.*, Supplemental Fig. S5

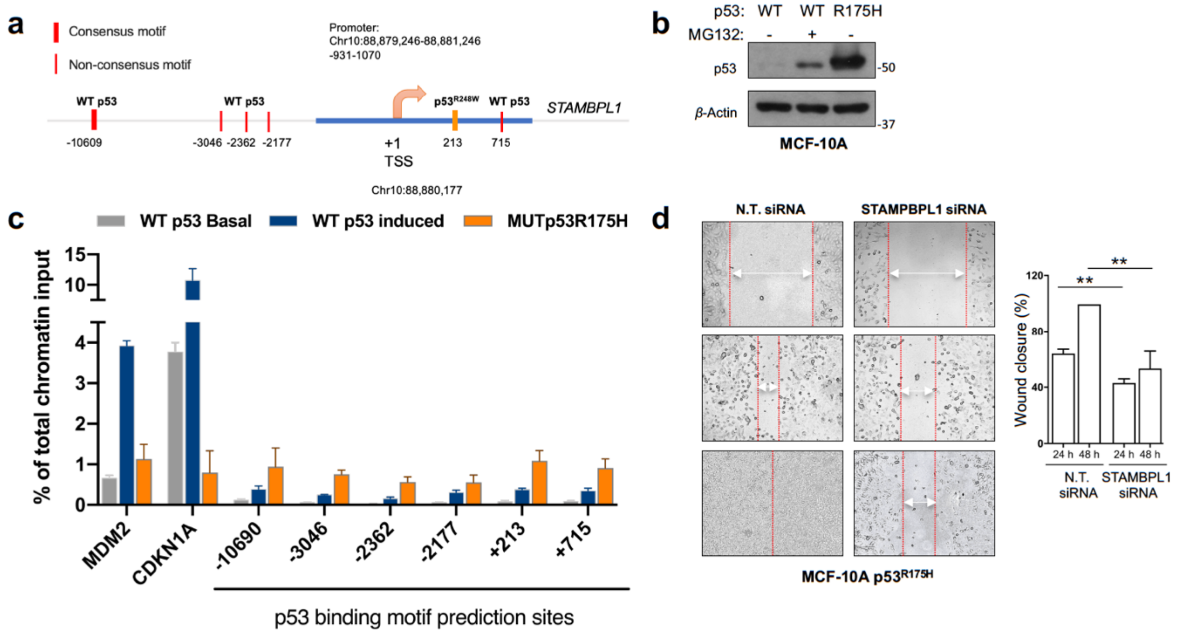


**Figure S5 legend. a** Putative p53 consensus or non-consensus binding elements motifs and locations in upstream promotor region of the human STAMBPL1 gene. TSS (transcription start site). **b** Western blot showing the expression of p53 in control or MG132 treated WT MCF10A cells, and p53 $^{R175H}$ mutant stable expressing MCF10A cells. $\beta$-Actin was used as a loading control for Western blot analysis. **c** ChIP-qPCR assay showing % enrichment of DNA fragments pulled using anti-p53 (DO-1) antibody over the total chromatin input. MDM2 and CDK1NA (p21) are shown as positive controls for the ChIP. 6 different regions on the STAMBPL1 (core and distal) promoter region was tested with unique primers for samples indicated in b. Enrichment over 1% is considered as positive. **d** Wound-healing assay in p53$^{R175H}$ MCF-10A cells transfected with NT or siRNA targeting STAMBPL1 for 48 h. Quantification is presented in normalized bar graphs (n=3). Statistical significance is shown over the control. *p<0.05 **p<0.001***p<0.0001 (Student's *t*-test). Data are presented as mean ± SD (n≥3).

Ambroise and Yu *et al.*, Supplementary Fig. S6: Original scans for Western blots.

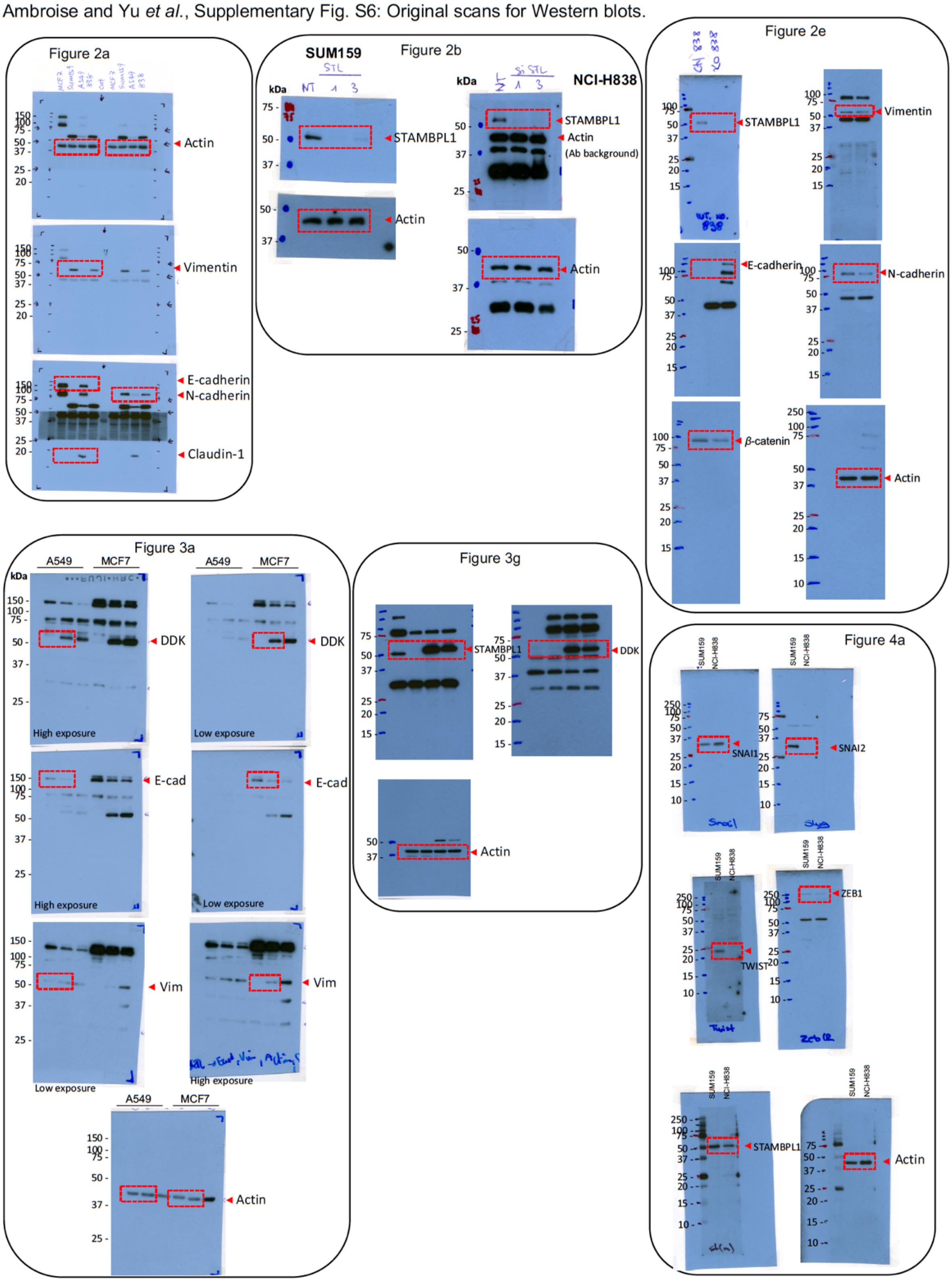

Ambroise and Yu *et al.*, Supplementary Fig. S7: Original scans for Western blots.

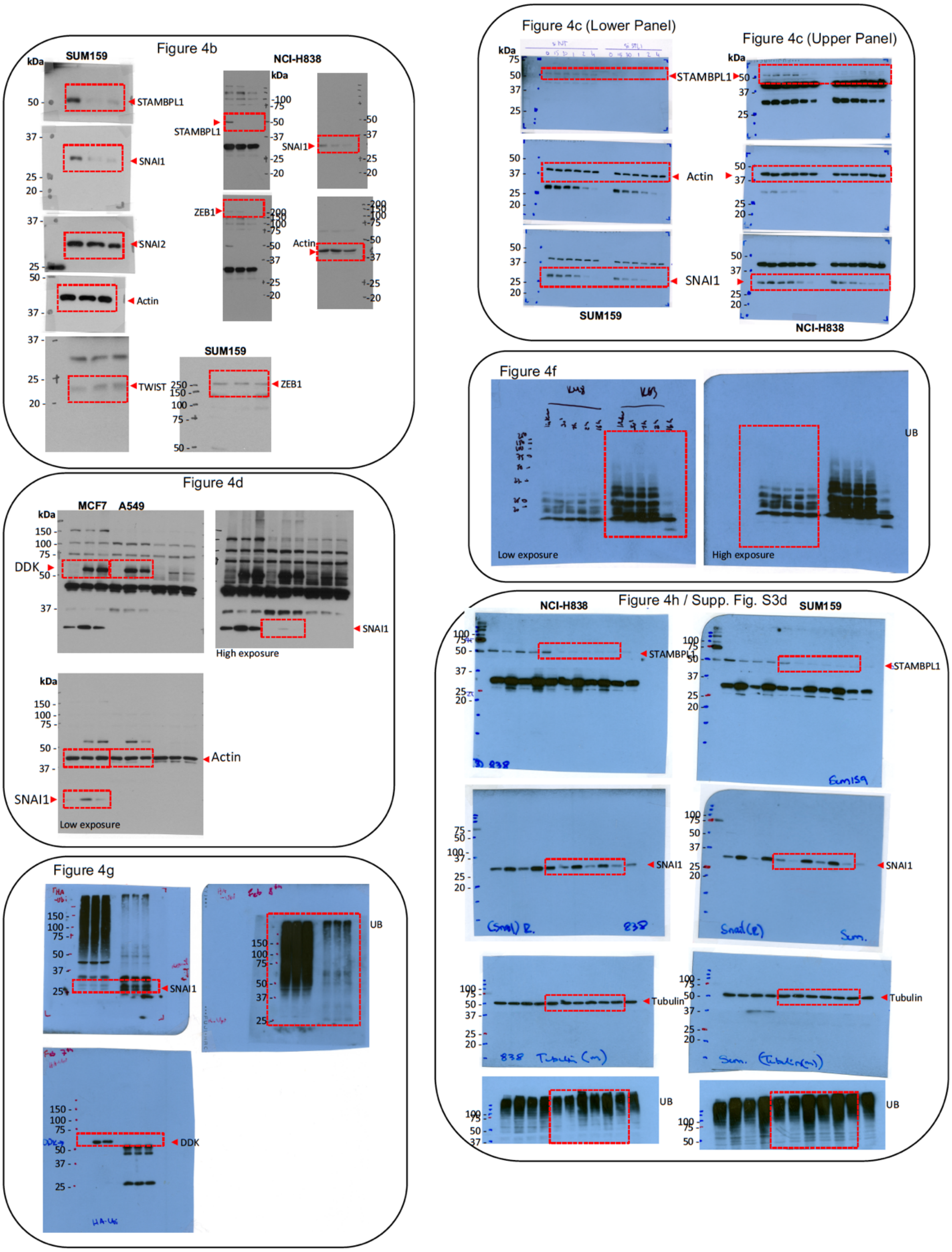

Ambroise and Yu *et al.*, Supplementary Fig. S8: Original scans for Western blots.

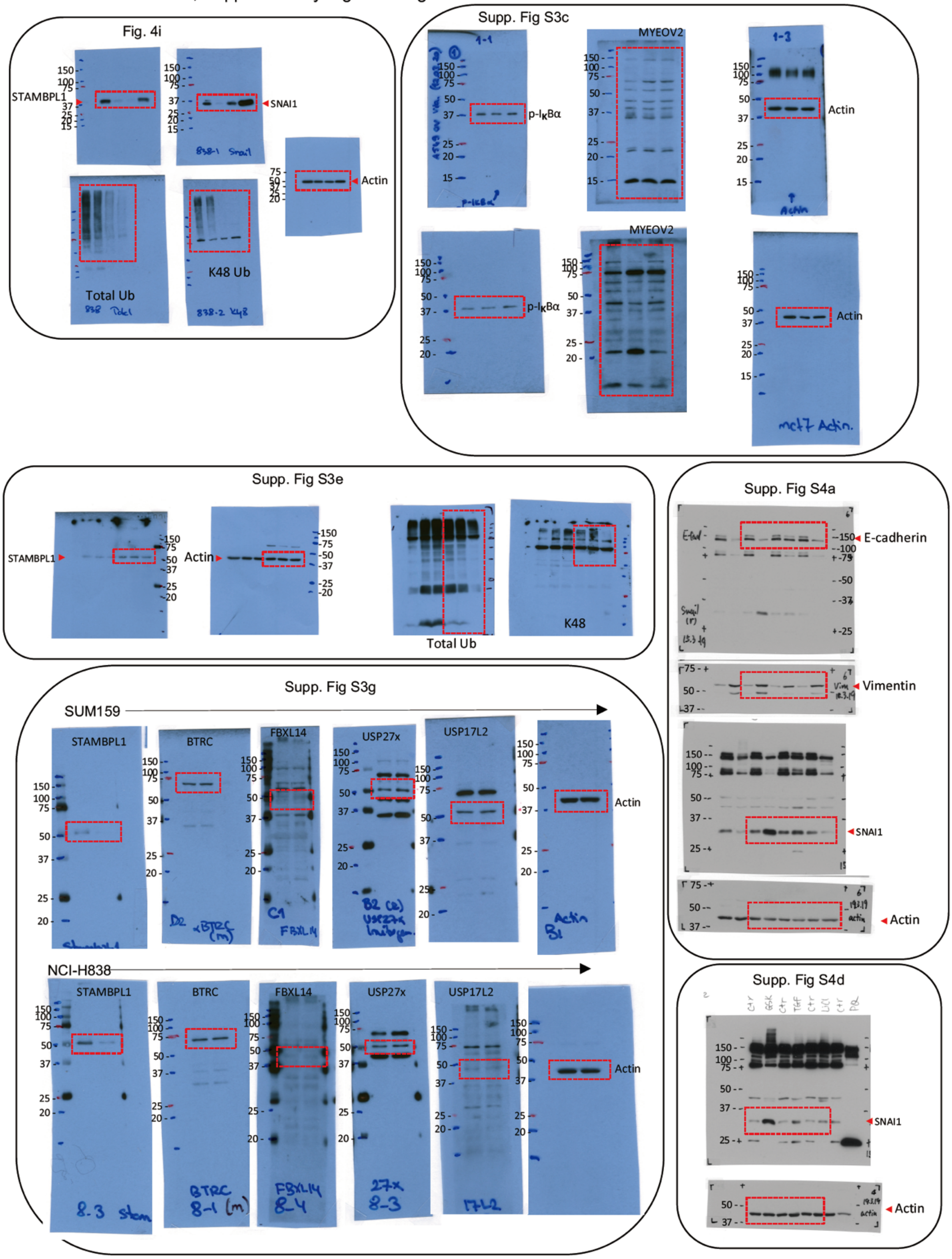

Ambroise and Yu *et al.*, Supplementary Fig. S9: Original scans for Western blots.

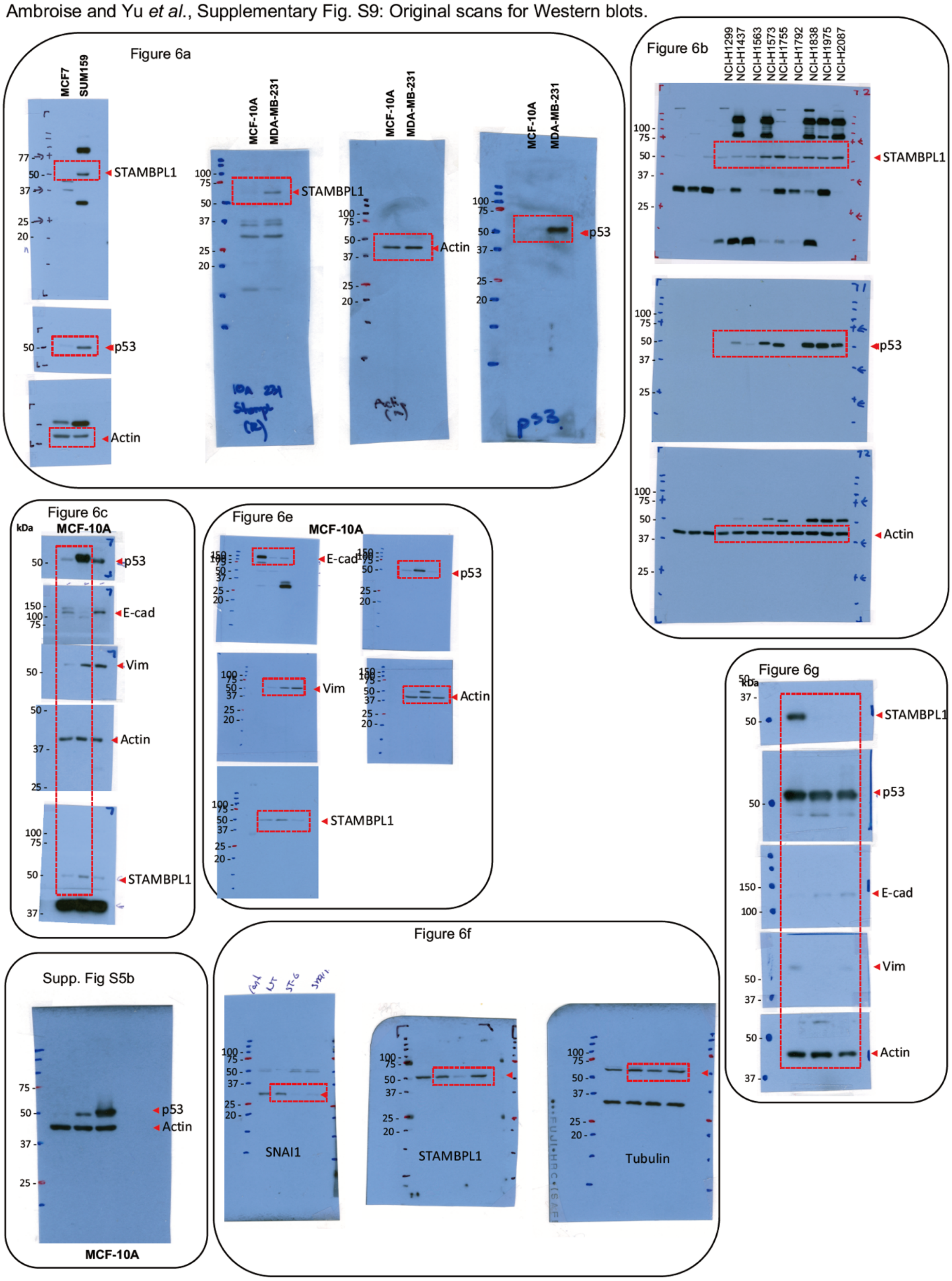

Supplement: Supplementary file 1 — Supplementary Material [file 41416_2020_972_MOESM1_ESM.pdf]
